# A genome-wide association study identifies key modulators of complement factor H binding to malondialdehyde-epitopes

**DOI:** 10.1073/pnas.1913970117

**Published:** 2020-04-22

**Authors:** Lejla Alic, Nikolina Papac-Milicevic, Darina Czamara, Ramona B. Rudnick, Maria Ozsvar-Kozma, Andrea Hartmann, Michael Gurbisz, Gregor Hoermann, Stefanie Haslinger-Hutter, Peter F. Zipfel, Christine Skerka, Elisabeth B. Binder, Christoph J. Binder

**Affiliations:** ^a^Department of Laboratory Medicine, Medical University of Vienna, 1090 Vienna, Austria;; ^b^Research Center for Molecular Medicine of the Austrian Academy of Sciences, 1090 Vienna, Austria;; ^c^Department of Medical Biochemistry, Faculty of Medicine, University of Sarajevo, 71000 Sarajevo, Bosnia and Herzegovina;; ^d^Department of Translational Research in Psychiatry, Max Planck Institute of Psychiatry, 80804 Munich, Germany;; ^e^Department of Infection Biology, Leibniz Institute for Natural Product Research and Infection Biology, 07745 Jena, Germany;; ^f^Central Institute for Medical and Chemical Laboratory Diagnosis, University Hospital Innsbruck, 6020 Innsbruck, Austria;; ^g^Institute for Microbiology, Friedrich Schiller University, 07743 Jena, Germany

**Keywords:** complement factor H, complement factor H-related protein 1, complement factor H-related protein 3, malondialdehyde, genome-wide association study

## Abstract

Genetic variants within complement factor H (CFH), a major alternative complement pathway regulator, are associated with the development of age-related macular degeneration (AMD) and other complementopathies. This is explained with the reduced binding of CFH or its splice variant factor H-like protein 1 (FHL-1) to self-ligands or altered self-ligands (e.g., malondialdehyde [MDA]-modified molecules) involved in homeostasis, thereby causing impaired complement regulation. Considering the critical role of CFH in inhibiting alternative pathway activation on MDA-modified surfaces, we performed an unbiased genome-wide search for genetic variants that modify the ability of plasma CFH to bind MDA in 1,830 individuals and characterized the mechanistic basis and the functional consequences of this. In a cohort of healthy individuals, we identified rs1061170 in *CFH* and the deletion of *CFHR3* and *CFHR1* as dominant genetic variants that modify CFH/FHL-1 binding to MDA. We further demonstrated that FHR1 and FHR3 compete with CFH for binding to MDA-epitopes and that FHR1 displays the highest affinity toward MDA-epitopes compared to CFH and FHR3. Moreover, FHR1 bound to MDA-rich areas on necrotic cells and prevented CFH from mediating its cofactor activity on MDA-modified surfaces, resulting in enhanced complement activation. These findings provide a mechanistic explanation as to why the deletion of *CFHR3* and *CFHR1* is protective in AMD and highlight the importance of genetic variants within the *CFH/CFHR3/CFHR1* locus in the recognition of altered-self in tissue homeostasis.

## Significance

Dysregulation of the alternative complement pathway due to impaired binding of complement factor H (CFH) to self-ligands or altered self-ligands (e.g. malondialdehyde [MDA]-modified molecules) involved in homeostasis can promote the development of complement-related diseases, such as age-related macular degeneration (AMD). We identified, in an unbiased GWAS approach, that common genetic variants within the CFH gene family (rs1061170 and the deletion of the complement factor H-related protein 1 and 3 genes [*CFHR3* and *CFHR1*]) act as major modulators of CFH recruitment and its ability to regulate complement on MDA-epitopes. These findings demonstrate the importance of the genetic status within the *CFH/CFHR3/CFHR1* locus in tissue homeostasis and provide a mechanistic explanation as to why deletion of *CFHR3/CFHR1* is protective in AMD development.

The complement system is an effector cascade of innate immunity that exerts key functions in host defense against invading infectious microbes (1). It also plays an important role in the maintenance of tissue homeostasis through immune surveillance and clearance of metabolic waste and dying cells (1–3). An impairment of critical complement functions caused by variations in genes for complement proteins has been associated with increased susceptibility to several inflammatory and autoimmune diseases, such as age-related macular degeneration (AMD), systemic lupus erythematosus (SLE), atypical hemolytic uremic syndrome (aHUS), and rheumatoid arthritis (RA) (4–6). Thus, tissue integrity requires efficient control of the complement cascade, which is achieved by regulators of complement activity like complement factor H (CFH).

CFH is a major inhibitor of the alternative complement pathway at the C3 level in both the soluble phase and on cellular surfaces (7). It is a highly abundant plasma glycoprotein composed of 20 short consensus repeat (SCR) domains arranged in a head-to-tail fashion. Moreover, its splice variant factor H-like protein 1 (FHL-1), consisting of the first seven SCRs of CFH, can be found in the circulation and tissues (1). The four N-terminal SCRs of CFH and FHL-1 are responsible for complement regulatory functions while the role of SCR6-8 is to recruit CFH and FHL-1, and of SCR19-20 to recruit CFH to complement vulnerable surfaces (8). Nonsynonymous polymorphisms and mutations within SCRs responsible for surface recognition of CFH and FHL-1 have been described as pathogenically relevant drivers of aHUS, C3 glomerulopathy (C3G), and AMD (9). This has been explained by an attenuated ability of CFH to bind self-ligands or altered self-ligands, such as double-stranded DNA, C-reactive protein (CRP), glycosaminoglycans (GAGs), sialic acid, and malondialdehyde (MDA)-epitopes (10–14). In line with this, we have shown that the AMD-associated minor allele of the single nucleotide polymorphism (SNP) rs1061170 (CFH-H402) within SCR7 impairs binding of CFH and FHL-1 to MDA-epitopes and its derivatives, malondialdehyde-acetaldehyde (MAA)-epitopes, present in the retina of AMD patients (14). Consequently, diminished neutralization of MDA-induced inflammation by CFH-H402 and decreased complement regulatory CFH cofactor activity on MDA-epitopes in retinal drusen can contribute to disease development.

MDA is a proinflammatory product of lipid peroxidation that contributes to the generation of altered self-structures that accumulate in many tissues associated with high levels of oxidative stress and tissue damage, which require prompt removal (15–18). It has been shown that MDA-epitopes mediate the clearance of damaged structures, such as oxidized low-density lipoproteins and dead cells (2). Various components of the innate immune system (e.g., scavenger receptors, natural immunoglobulin M (IgM) antibodies, CFH, and FHL-1) either directly or indirectly facilitate this process (14, 19–21). On the other hand, an impaired removal of MDA-carrying metabolic waste will promote inflammation (14, 19, 22).

Considering the role of MDA as a marker of dangerous self-structures and the critical role of CFH and FHL-1 in inhibiting complement activation on MDA-decorated surfaces, we searched in an unbiased, genome-wide approach for genetic variants that could modify this function of CFH in plasma and assessed the functional consequences thereof.

## Results

### Plasma CFH/FHL-1 Binding to MDA-Epitopes Displays High Variability.

To assess the individual variation of plasma CFH binding to MDA in our cohort, we developed an enzyme-linked immunosorbent assay (ELISA) exclusively detecting CFH and its splice variant FHL-1 (CFH/FHL-1), but not factor H-related proteins (FHRs), using a monoclonal antibody that recognizes SCR5 of CFH/FHL-1 (*SI Appendix*, Fig. S1 *A*–*C*).

CFH/FHL-1 binding to MDA-modified bovine serum albumin (MDA-BSA) and CFH/FHL-1 concentrations were measured in plasma of 934 unrelated healthy individuals and of 896 unrelated patients with major depression disorder (MDD). Both cohorts used were age- and gender-matched, and the majority of recruited subjects were Caucasians of German origin (23). The general characteristics and measured parameters of the study cohorts are summarized in Table 1. Because healthy individuals and the MDD group were comparable with respect to demographic characteristics and the CFH measurements (CFH/FHL-1 binding and plasma concentration), all subjects were considered as one integrated cohort of 1,830 subjects where appropriate. CFH/FHL-1 concentrations in plasma of all subjects was skewed to the right (*SI Appendix*, Fig. S2*A*) while plasma CFH/FHL-1 binding to MDA-BSA displayed a normal distribution, albeit with high individual variability within the cohort (*SI Appendix*, Fig. S2*B*).

**Table 1.** Characteristics and main measured parameters of the study cohorts

	Healthy individuals (*n* = 934)	Patients with major depression (*n* = 896)	Difference between healthy individuals and patients, *P* value	Integrated cohort (*n* = 1,830)
Age, y; mean (± SD)	50.9 (±13.9)	51.1 (13.8)	0.902*	51.0 (±13.9)
Female, no. (percentage)	632 (67.6%)	548 (66.7%)	0.683^†^	1,180 (67.2%)
CFH/FHL-1 binding to MDA-BSA, RLU/100 ms; median (interquartile range)	24,222 (18,610–29,786)	24,836 (19,689–29,879)	0.126*	24,550 (19,044–29,826)
CFH/FHL-1 plasma concentration, µg/mL; median (interquartile range)	411.0 (323.7–537.6)	416.8 (319.1–535.9)	0.468*	414.1 (320.1–536.1)

* Differences tested with Mann–Whitney *U* test.

^†^ Differences tested with Fisher’s exact test.

### A Genome-Wide Association Study (GWAS) Identifies rs1061170 as the Most Potent Genetic Modulator of CFH/FHL-1 Binding to MDA.

**Fig. 1.**
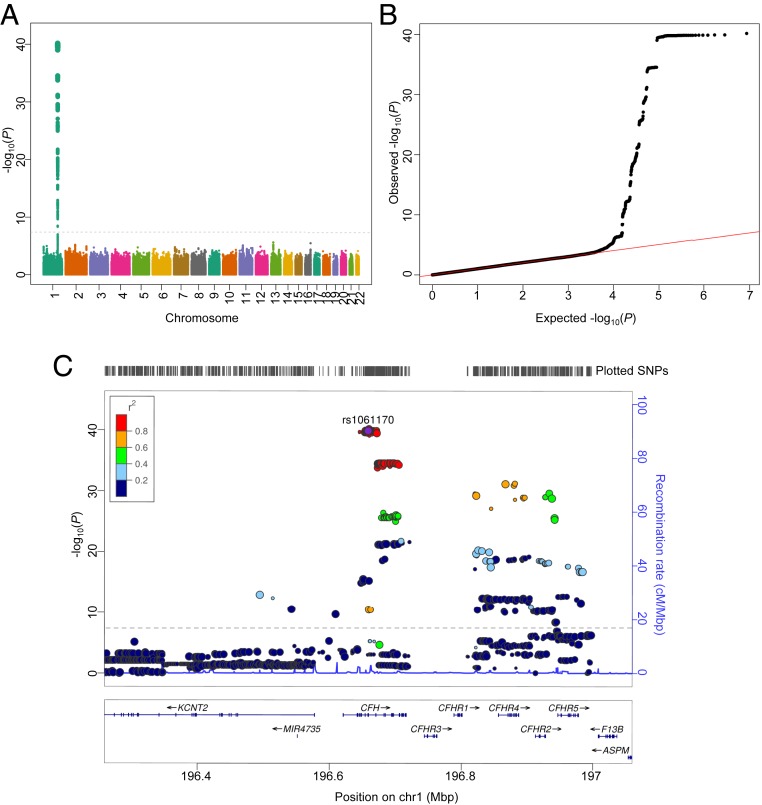
GWAS of plasma CFH/FHL-1 binding to MDA-BSA in the integrated cohort (*n* = 1,830). (*A*) Manhattan plot showing −log_10_(*P*) values for all SNPs. The plot includes genotyped and imputed unpruned data. Significant SNPs are located in the 1q31.3 region. (*B*) Quantile–quantile (QQ) plot for the genotyped and imputed SNP interaction with CFH/FHL-1 binding to MDA-BSA. Observed *P *values (black dots) are plotted against the expected* P *values if no association is assumed (full red line). (*C*) LocusZoom plot showing unpruned SNPs associated with CFH/FHL-1 binding to MDA-BSA with −log_10_(*P*) values based on an analysis adjusted for age, sex, case-control status, and first two principal components to correct for population stratification. Color coding represents linkage disequilibrium r^2^ values of neighboring SNPs to the leading exonic SNP rs1061170. Gray dashed line set at GWAS significance threshold at −log_10_(*P*) = 7.3 (*P* = 5 × 10^−8^) (*A* and *C*). Mbp, mega base pairs.

In order to identify factors that modulate CFH/FHL-1 binding to MDA-BSA, we performed a GWAS in our integrated cohort of 1,830 individuals. SNPs clustered in the 1q31.3 region on chromosome (chr) 1 displayed the strongest genome-wide significant association (*P* < 5 × 10^−8^) for CFH/FHL-1 binding to MDA-epitopes in an analysis adjusted for age, sex, case-control status, and the first two principal components to correct for population stratification (Fig. 1 *A* and *B*). Of these, rs10801556, an intronic variant in *CFH* (*P* = 7.03 × 10^−41^), was the top hit. This SNP is in complete linkage disequilibrium (LD) (D′ = 1, *r*^2^ = 1, 1000 Genomes, phase 3, European population) with the missense exonic variant rs1061170 (Y402H), having an association *P* value = 1.45 × 10^−40^ (Fig. 1*C*). Each copy of the minor allele of rs1061170 C decreased CFH/FHL-1 binding to MDA-BSA by β = −4221.1 ± 304.8 (SD) relative light units (RLU)/100 ms. When all top hit SNPs were additionally conditioned on rs1061170, *P* values of the neighboring SNPs significantly decreased, indicating that *P* values were driven by associations to rs1061170 (*SI Appendix*, Fig. S3 *A*–*C*). After this adjustment, the intergenic variant rs371960809 located downstream of the *CFH* gene had the highest, although subthreshold, significance *P* value of 1.81 × 10^−7^. Of note, before adjustment, this SNP had a GWAS *P* value of 2.53 × 10^−22^ and was one of the top hits. Although we have previously shown that SCR19-20 of CFH contain an MDA binding site as well (14), we did not find an association between CFH/FHL-1 binding to MDA-epitopes and SNPs located in the exons 21 to 22 of the *CFH* gene, demonstrating the importance of genetic variants within SCR7 in the recognition and binding of MDA-epitopes. With regard to the plasma CFH/FHL-1 concentrations, the SNP that most strongly associated with CFH/FHL-1 levels in our integrated cohort was the intergenic variant rs10784193, located in chromosome 12 (*P* = 3.14 × 10^−6^; minor allele A β = −33.1 ± 7.0 [SD] µg/mL) (*SI Appendix*, Fig. S4 *A*–*C*). Notably, this SNP did not alter CFH/FHL-1 binding to MDA-BSA (*SI Appendix*, Fig. S4*D*).

**Fig. 2.**
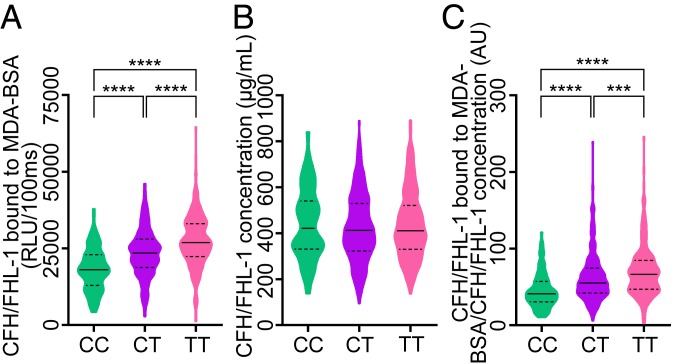
Association between the rs1061170 genotype and (*A*) CFH/FHL-1 binding to MDA-BSA, (*B*) CFH/FHL-1 concentration, and (*C*) CFH/FHL-1 binding to MDA-BSA normalized to the CFH/FHL-1 concentration in plasma of healthy individuals (*n* = 934). (*A*) CC, *n* = 120; CT, *n* = 367; TT, *n* = 280. (*B*) CC, *n* = 120; CT, *n* = 362; TT, *n* = 279. (*C*) CC, *n* = 119; CT, *n* = 361; TT, *n* = 278. Data are presented as violin plot, where dashed lines represent quartiles, full lines represent median, and widths represent the number of individuals with the same value of the measured parameter. Statistical differences were tested using the Kruskal–Wallis test, with Dunn’s multiple comparison. ***P value ≤ 0.001, ****P value ≤ 0.0001. AU, arbitrary units.

To exclude any potential biases due to the presence of MDD, we evaluated our GWAS using only the cohort of healthy individuals (*n* = 934). Similar results were obtained, with rs1061170 being the top hit SNP (*P* value = 3.31 × 10^−22^; minor allele C β = −4,250.0 ± 420.3 [SD] RLU/100 ms) (*SI Appendix*, Fig. S5 *A*–*C*). Moreover, after adjustment for rs1061170, rs371960809 was the most significant hit (*P* = 1.72 × 10^−6^), confirming the results from the integrated cohort. Consistent with our previously reported findings in the AMD cohort, the nonsynonymous Y402H variant significantly influenced CFH/FHL-1 binding to MDA-BSA in healthy individuals. Plasma samples of individuals homozygous for the CFH-H402 variant (CC) displayed the lowest CFH/FHL-1 binding to MDA-BSA (Fig. 2*A*). The strongest association with plasma CFH/FHL-1 concentrations in healthy individuals was found with rs2712503, an intronic variant of the long intergenic nonprotein coding RNA 2388 on chromosome 12 (*P* = 3.25 × 10^−7^; minor allele A β = 72.0 ± 13.9 [SD] µg/mL). Plasma CFH/FHL-1 concentrations were comparable when stratified according to the Y402H genotype, and, after normalization for individual CFH/FHL-1 concentrations, the differences in CFH/FHL-1 binding to MDA-BSA according to the Y402H genotype did not change (Fig. 2 *B* and *C*). These data confirm that the differences in the ability of CFH/FHL-1 to bind to MDA-BSA in our assay depend on genetic variants within *CFH*, but not on CFH/FHL-1 levels.

### A GWAS Reveals a Cluster of Genetic Variants within *CFHR* Genes as Modulators of CFH/FHL-1 Binding to MDA-Epitopes.

**Fig. 3.**
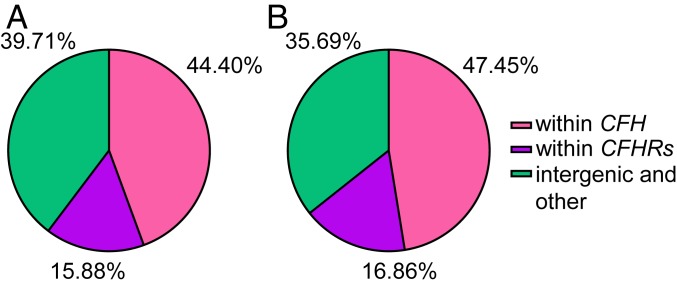
Location of SNPs with GWAS significance within the *CFH-CFHR* cluster. (*A*) Location of SNPs in the integrated cohort (*n* = 1,830) and (*B*) cohort of healthy individuals (*n* = 934).

Interestingly, in all three groups of healthy individuals stratified according to their rs1061170 status, we observed high variability of CFH/FHL-1 binding to MDA-BSA (interquartile range [IQ] range for carriers of the CC variant, 10,001 RLU/100 ms; CT variant, 9,226 RLU/100 ms; and TT variant, 10,705 RLU/100 ms). One plausible explanation for such variability is that additional factors—irrespective of the rs1061170 genotype—exist, which could influence CFH/FHL-1 binding to MDA-epitopes. In line with this, before and after adjustment for rs1061170, an SNP cluster outside *CFH* that had the most significant genetic association with CFH/FHL-1 binding to MDA-BSA in the integrated and the healthy cohort was located within *CFHR2* (chr1: 196,912,888 base pairs [bp] to 196,928,356 bp), *CFHR4* (chr1: 196,857,144 bp to 196,887,843 bp), and *CFHR5* (chr1: 196,946,667 bp to 196,978,804 bp). Notably, out of all SNPs with genome-wide significance, 15.88% in the integrated cohort and 16.86% in the healthy individuals were located within these genes (Fig. 3 *A* and *B*). Although we have previously demonstrated that FHR5 also binds to MDA-epitopes, all 17 SNPs within the *CFHR5* gene locus had the lowest GWAS *P* value compared to all other SNPs, after adjustment for rs1061170, and were located in intronic regions. Therefore, these SNPs were excluded from further analyses (*SI Appendix*, Fig. S3*C*). Next, we tested if FHR2 and FHR4 would also bind to MDA-modified proteins. Neither FHR2 nor FHR4 bound to MDA-epitopes irrespective of the carrier protein (*SI Appendix*, Fig. S6). In contrast to FHR2 and FHR4, FHR1 and FHR3 have been significantly associated with complementopathies, such as AMD and aHUS. However, *CFHR3* and *CFHR1* gene variants were neither covered in the SNP array used nor could they be imputed successfully. Therefore, the effect of variants within these two genes on the ability of CFH/FHL-1 to bind MDA-epitopes could not be analyzed in the same manner. Because copy number variations (CNVs) within *CFHR3* and *CFHR1* are the most common genetic variations of these genes and determine plasma levels of these proteins (24–29), we performed a multiplex ligation probe amplification assay (MLPA) to detect CNVs present within the *CFHR3-CFHR1* loci of the cohort of healthy individuals.

### CNVs in *CFHR3* and *CFHR1* Influence CFH/FHL-1 Binding to MDA-Epitopes Irrespectively of the rs1061170 Genotype.

The results of the MLPA analysis in the cohort of healthy individuals (*n* = 961) are presented in Table 2. The allele frequency of *CFHR3* and *CFHR1* deletions was 20.9% while the frequency of homozygous *CFHR3* and *CFHR1* deletions (del/del *CFHR3* and *CFHR1*) was 4.2%. The allele frequency of the *CFHR3* and *CFHR1* deletions did not deviate from the Hardy–Weinberg equilibrium (*P* = 0.39) and is in line with previous reports from other European populations (26).

**Table 2.** Total number and frequency of CNVs in *CFHR3* and *CFHR1* genes detected by MLPA in healthy individuals (*n* = 961)

Genotype	No.	Percentage (%)
+/+ *CFHR3* and *CFHR1*	582	60.6
+/del *CFHR3* and *CFHR1*	317	33.0
del/del *CFHR3* and *CFHR1*	40	4.2
+/+ *CFHR3*, +/del *CFHR1*	13	1.4
+/+ *CFHR3*, 3x *CFHR1*	3	0.3
+/del *CFHR3*, +/+ *CFHR1*	2	0.2
+/del *CFHR3*, del/del *CFHR1*	2	0.2
del/del *CFHR3*, +/del *CFHR1*	2	0.2

**Fig. 4.**
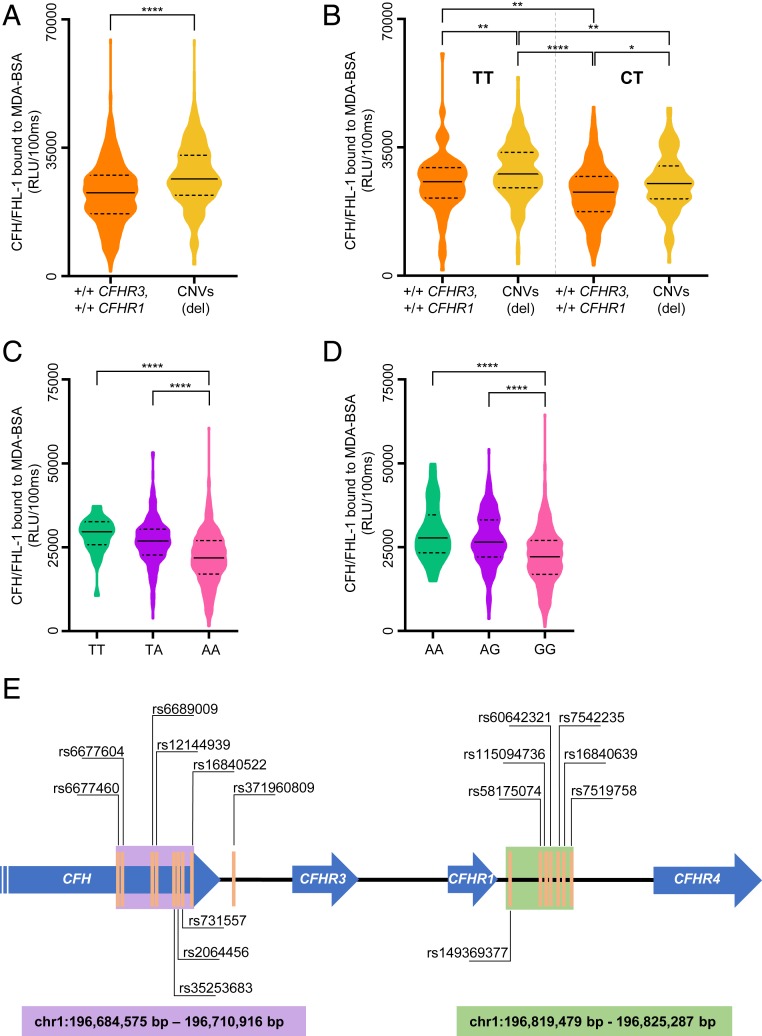
Association between *CFHR3* and *CFHR1* CNVs (del) and CFH/FHL-1 binding to MDA-BSA in plasma of healthy individuals (*n* = 934) and schematic representation of the proxy SNPs for *CFHR3* and *CFHR1* deletion on chromosome 1. (*A* and *B*) Association between *CFHR3* and *CFHR1* CNVs (del) and CFH/FHL-1 binding to MDA-BSA in (*A*) healthy individuals that were +/+ *CFHR3*, *CFHR1* (*n* = 564), or CNVs (del) (*n* = 350) and (*B*) healthy individuals with the rs1061170 TT (+/+ *CFHR3, CFHR1 n* = 113 vs. CNVs [del] *n* = 164) and CT genotype (+/+ *CFHR3, CFHR1 n* = 244 vs. CNVs [del] *n* = 114). The CNVs (del) group includes carriers of +/del *CFHR3* and *CFHR1,* del/del *CFHR3* and *CFHR1,* +/del *CFHR3* and del/del *CFHR1*, del/del *CFHR3* and +/del *CFHR1*. The distribution of the rs1061170 genotypes in (*A*) is 116 CC and 244 CT for the +/+*CFHR3*, *CFHR1* group and 2 CC and 114 CT for the CNVs (del) group. (*C* and *D*) CFH/FHL-1 binding to MDA-BSA in healthy individuals according to (*C*) rs371960809 (individuals homozygous for the minor allele [TT], *n* = 33; heterozygous for the minor allele [TA], *n* = 244; homozygous for the major allele [AA], *n* = 488) and (*D*) rs6677604 genotype (individuals homozygous for the minor allele [AA], *n* = 33; heterozygous for the minor allele [AG], *n* = 250; homozygous for the major allele [GG], *n* = 489). (*E*) Schematic representation of the genomic position of proxy SNPs for *CFHR3* and *CFHR1* deletions on chromosome 1. Genes are represented as blue arrows and selected SNPs are represented as orange vertical lines. SNP clusters of proxy SNPs for *CFHR3* and *CFHR1* deletion are located within *CFH* (highlighted with a purple box) and the region of the *CFHR4* pseudogene (highlighted with a green box). Data in (*A*–*D*) presented as violin plot where dashed line represents quartiles, full line represents median and width represents the number of individuals with the same value of the measured parameter. Statistical differences were tested using the Mann–Whitney *U* test (*A*) or Kruskal–Wallis test with Dunn’s multiple comparison (*B–D*). *P value ≤ 0.05, **P value ≤ 0.01,****P value ≤ 0.0001.

In order to evaluate the influence of detected CNVs of *CFHR3* and *CFHR1* on the ability of CFH/FHL-1 to bind MDA-BSA, the CNV status was associated with CFH/FHL-1 binding to MDA-epitopes. Due to the small number of individuals with gene multiplications (*n* = 3), CNVs were exclusively considered as an absence of at least one allele of both *CFHR3* and *CFHR1* (CNVs [del]) in subsequent analyses. We could show that individuals with at least one deleted DNA fragment containing both *CFHR3* and *CFHR1* genes had higher binding of CFH/FHL-1 to MDA-BSA (Fig. 4*A*), indicating that decreased levels of FHR1 and FHR3 lead to enhanced CFH/FHL-1 binding to MDA-BSA. To test whether this effect is dependent on the rs1061170 genotype, the LD between rs1061170 and *CFHR3* and *CFHR1* deletions was examined. There was no LD between rs1061170 and CNVs in *CFHR3* and *CFHR1* (r = −0.408) in our cohort, which is in concordance with literature (29). This suggests that the presence of FHR1 and FHR3 inhibits binding of CFH-Y402 and CFH-H402 variants of CFH/FHL-1 to MDA-epitopes. Indeed, in both carriers of the TT (homozygous CFH-Y402) as well as the CT (heterozygous CFH-H402) rs1061170 genotype, higher CFH/FHL-1 binding to MDA-BSA was detected already when one fragment of DNA containing both *CFHR3* and *CFHR1* was deleted (CNVs [del] = +/del *CFHR3* and *CFHR1*, del/del *CFHR3* and *CFHR1,* +/del *CFHR3* and del/del *CFHR1*, del/del *CFHR3* and +/del *CFHR1*) (Fig. 4*B*). Thus, the effect of CNVs (del) in *CFHR3* and *CFHR1* on CFH/FHL-1 binding to MDA is independent of the rs1061170 status, and both the rs1061170 genotype as well as the absence or presence of FHR1 and FHR3 determine the overall ability of CFH/FHL-1 to be recruited to MDA-covered surfaces.

In addition, when our MLPA data were associated with the top hit SNPs for CFH/FHL-1 binding to MDA-BSA within the healthy cohort, we identified a group of 16 SNPs that are in high LD (*r* ≥ 0.945) with deletions of *CFHR3* and *CFHR1*. The SNPs rs16840522, rs16840639, rs7519758, rs7542235, rs6677604, and rs12144939 that showed the highest correlation with deletions of *CFHR3* and *CFHR1* have been previously described (27, 30–33). Moreover, a high correlation was observed for rs371960809, rs115094736, rs58175074, rs149369377, rs60642321, rs2064456, rs35253683, rs6677460, rs6689009, and rs731557 (Table 3), suggesting that they could serve as proxies for *CFHR3* and *CFHR1* deletions. As expected, the genotype of all above mentioned SNPs influences CFH/FHL-1 binding to MDA-BSA, as demonstrated for rs371960809 (LD *r* = 0.954) and rs6677604 (LD *r* = 0.945) (Table 3 and Fig. 4 *C* and *D*). This effect was independent of the rs1061170 status as rs1061170 has a low LD with these two SNPs (r = −0.427, r = −0.424, respectively). Additionally, stratification of individuals in the integrated cohort of 1,830 individuals, according to the rs1061170 and rs371960809 status (as proxy for *CFHR3* and *CFHR1* deletions), confirmed our observation (*SI Appendix*, Fig. S7). Interestingly, these 16 SNPs formed two distinct clusters: one comprised of eight intronic SNPs within *CFH* (chr1: 196,684,575 bp to 196,710,916 bp) and seven SNPs located in the segmental duplication A region flanking the break point for deletion of *CFHR3* and *CFHR1* (chr1: 196,819,479 bp to 196,825,287 bp) (Fig. 4*E*) (34). Our data demonstrate that rs1061170 and *CFHR3* and *CFHR1* deletion are the most significant genetic variants that modify the ability of CFH/FHL-1 to bind MDA-epitopes.

**Table 3.** SNP alleles spanning 2 Mbp from del/del *CFHR3* and *CFHR1* with the highest correlation with the deletion

SNP_minor allele	r^2^ with del/del *CFHR3* and *CFHR1*
rs16840522_C	0.954
rs371960809_T	0.954
rs115094736_G	0.953
rs58175074_G	0.953
rs149369377_G	0.952
rs16840639_C	0.951
rs7519758_T	0.951
rs60642321_G	0.951
rs7542235_G	0.949
rs12144939_T	0.945
rs2064456_G	0.945
rs35253683_A	0.945
rs6677460_G	0.945
rs6677604_A	0.945
rs6689009_G	0.945
rs731557_A	0.945

Mbp, mega base pairs.

### FHR1 and FHR3 Specifically Bind to MDA-Epitopes.

**Fig. 5.**
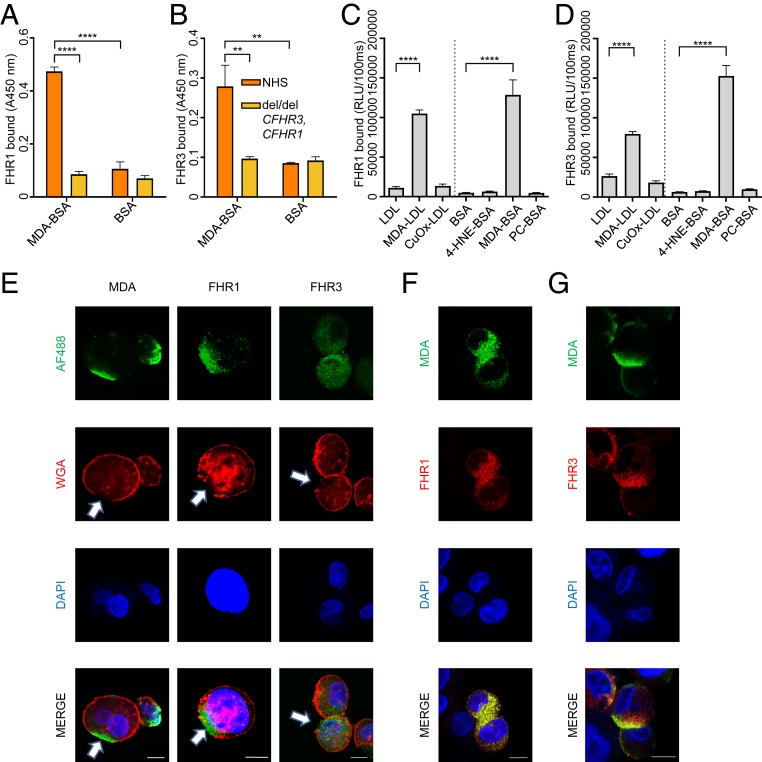
Binding of FHR1 and FHR3 to MDA-epitopes. Binding of serum-derived (*A*) FHR1 and (*B*) FHR3 to coated MDA-BSA and sham-treated BSA (BSA). Bars represent mean ± SEM values of triplicate determinations, and a representative ELISA of two is shown. Binding of recombinant (*C*) FHR1 and (*D*) FHR3 to coated oxidation-specific epitopes. Bars represent mean ± SEM values of quadruplicate determinations with data pooled from two independent experiments. (*E*) Fluorescence microscopy demonstrating MDA-enrichment on necrotic HUVECs (*Left Column*). Binding of FHR1 and FHR3 to necrotic HUVECs on damaged membrane areas (*Middle* and *Right Columns*). Sites on cellular membranes with lost integrity are highlighted with arrows. (*F*) Fluorescence microscopy demonstrating colocalization (yellow) between MDA-epitopes (green) and FHR1 (red) on necrotic HUVECs. (*G*) Fluorescence microscopy demonstrating colocalization (yellow) between MDA-epitopes (green) and FHR3 (red) on necrotic HUVECs. (*A*–*D*) Statistical differences were tested by one-way ANOVA, with Tukey’s multiple comparison. **P value ≤ 0.01,****P value ≤ 0.0001. (Scale bars: *E*–G, 10 µm.) AF488, Alexa Fluor 488; DAPI, 4′,6-diamidino-2-phenylindole; NHS, normal human serum.

In order to elucidate our finding that variations within the region of *CFHR3* and *CFHR1* can modulate the binding of CFH/FHL-1 to MDA-epitopes, we assessed the binding of serum-derived FHR1 and FHR3 to MDA-BSA. We could demonstrate that FHR1 and FHR3 present in human serum bound to MDA-BSA while this was not seen when serum of a del/del *CFHR3* and *CFHR1* individual was used (Fig. 5 *A* and *B*). These data were confirmed for FHR1 using plasma of +/+ *CFHR3* and *CFHR1* individuals and del/del *CFHR3* and *CFHR1* individuals with the same rs1061170 genotype and similar CFH concentrations, demonstrating exclusive binding of plasma-derived FHR1 to MDA-epitopes but not to other oxidation-specific epitopes (OSEs) (*SI Appendix*, Fig. S8*A*). Moreover, recombinant FHR1 and FHR3 bound exclusively to MDA-modified low-density lipoprotein (MDA-LDL) and MDA-modified BSA, but not to phosphorylcholine (PC)- and 4-hydroxynonenal (HNE)-modified BSA or copper sulfate–oxidized LDL (CuOx-LDL), which is replete with PC-carrying oxidized phospholipids (Fig. 5 *C* and *D*). In line with this, we could show that biotinylated MDA-BSA also bound to coated FHR1 or FHR3 in a concentration-dependent manner (*SI Appendix*, Fig. S8 *B* and *C*). Furthermore, the affinity of the CFH::MDA, FHR1::MDA, and FHR3::MDA interaction was compared using microscale thermophoresis with both binding partners in fluid phase. The binding affinity for MDA-epitopes for CFH was 2.44 µM, for FHR1 was 0.86 µM, and for FHR3 was 3.48 µM (Table 4). Thus, FHR1 displayed a threefold and fourfold higher affinity for MDA-epitopes compared to CFH and FHR3, respectively.

**Table 4.** Measured affinity for CFH, FHR1, and FHR3 binding to MDA-covered surface

Protein	KD, µM
CFH	2.44
FHR1	0.86
FHR3	3.48

KD, equilibrium dissociation constant.

One of the major carriers of MDA-epitopes in vivo are apoptotic and necrotic cells (20, 35). CFH as well as FHR5 have been found to bind to MDA-epitopes on necrotic cells (14, 21). Therefore, we also tested the binding of recombinant FHR1 and FHR3 to necrotic human umbilical vein endothelial cells (HUVECs) by fluorescence microscopy. The cell membrane structure was detected with wheat germ agglutinin (WGA), and the presence of the MDA-epitopes was detected using MDA-specific antibodies (LR04 or NA17). On necrotic HUVECs, areas with membrane damage were enriched in MDA-epitopes (Fig. 5 *E*, *Left Column*), and binding of FHR1 followed the same pattern (Fig. 5 *E*, *Middle Column* and *SI Appendix*, Fig. S9*A*). Furthermore, we could demonstrate exclusive colocalization of FHR1 and MDA-epitopes on necrotic cells (Fig. 5*F*). FHR3 also colocalized with MDA-epitopes but showed a more diffuse pattern on the cell surface, with a slight enrichment for MDA-positive areas (Fig. 5 *E*, *Right Column*, Fig. 5*G*, and *SI Appendix*, Fig. S9*B*) (36). These data demonstrate that both FHR1 and FHR3 identify MDA-epitopes at sites of membrane damage, albeit with different affinities.

### FHR1 Competes with CFH/FHL-1 for MDA Binding and Affects CFH-Mediated Complement Regulation on MDA-Decorated Surfaces.

**Fig. 6.**
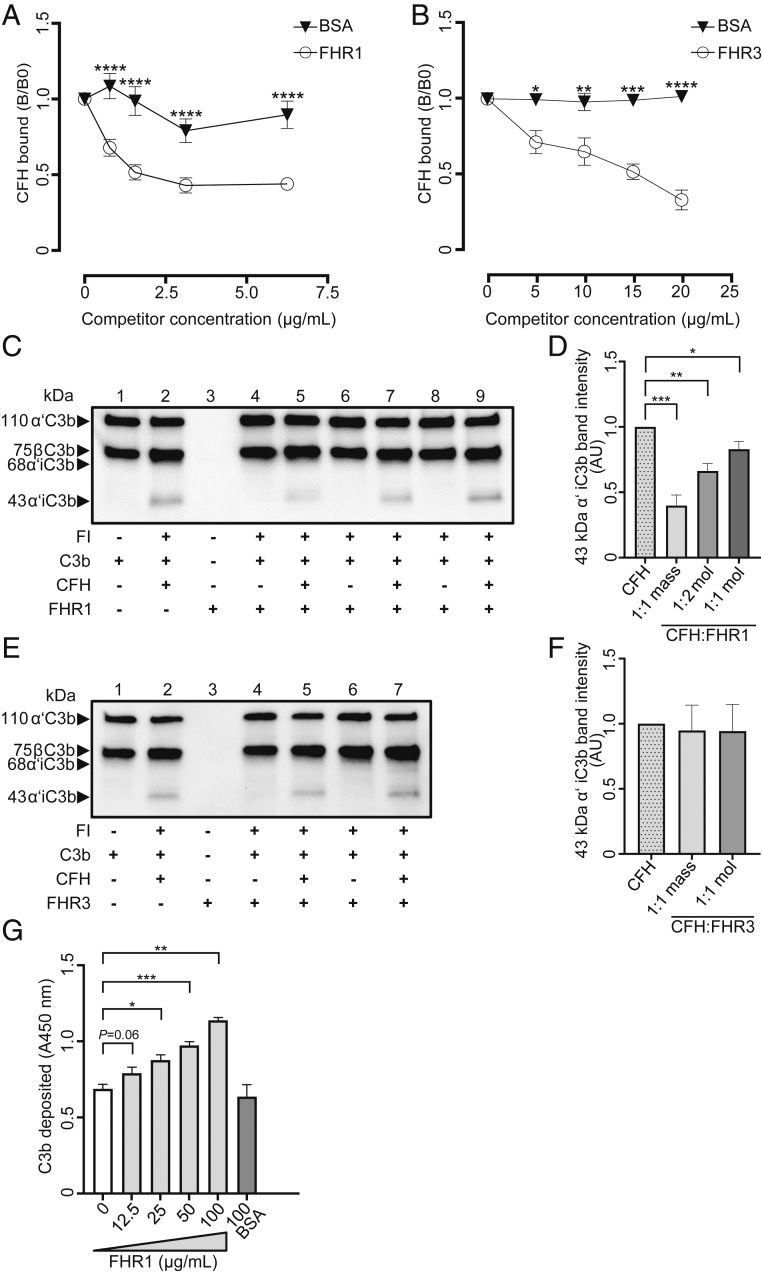
Functional consequences of FHR1 and FHR3 binding to MDA-surfaces. Competition assay of binding of biotinylated CFH to coated MDA-BSA with increasing concentrations of (*A*) FHR1 and (*B*) FHR3 or BSA as control. Each data point represents the mean ± SEM of triplicate determinations of (*A*) three and (*B*) two independent experiments. Data are presented as ratios between CFH binding in the presence of competitor divided by the CFH binding in the absence of competitor (B/B0). (*C*–*F*) CFH cofactor activity assays. (*C*) A representative immunoblot showing C3b degradation products generated in MDA-coated wells in the presence of the indicated proteins. Lane 2 contains 10 µg/mL CFH. Lane 4 contains 10 µg/mL FHR1. Lane 5 contains a 1:1 mass ratio of CFH and FHR1 (10 µg/mL each). Lane 6 contains 144 nM FHR1. Lane 7 contains a 1:2 molar ratio of CFH and FHR1. Lane 8 contains 72 nM FHR1. Lane 9 contains a 1:1 molar ratio of CFH and FHR1 (72 nM each). (*D*) Densitometric analysis of the bands representing the 43-kDa iC3b degradation products as shown in *C*. Data are from seven independent experiments. Bars represent the mean ± SEM fold differences in density of the 43-kDa iC3b bands compared to the condition shown in lane 2 (CFH only). (*E*) A representative immunoblot showing C3b degradation products generated in MDA-coated wells in the presence of the indicated proteins. Lane 2 contains 10 µg/mL CFH. Lane 4 contains 10 µg/mL FHR3. Lane 5 contains a 1:1 mass ratio of CFH and FHR3 (10 µg/mL each). Lane 6 contains 72 nM FHR3. Lane 7 contains a 1:1 molar ratio of CFH and FHR3 (72 nM each). (*F*) Densitometric analysis of the bands representing the 43-kDa iC3b degradation products as shown in *E*. Data are from eight independent experiments. Bars represent the mean ± SEM fold differences in density of the 43-kDa iC3b bands compared to the condition shown in lane 2 (CFH only). (*G*) ELISA for deposition of C3b from del/del *CFHR3* and *CFHR1* serum spiked with increasing concentrations of recombinant FHR1 or BSA (negative control) to MDA-coated wells. Bars represent mean ± SEM values of triplicate determinations with data pooled from four independent experiments. Statistical differences were tested by two-way ANOVA with Sidak's multiple comparison (*A* and *B*) and Mann–Whitney *U* test (*D*, *F*, and *G*). *P value ≤ 0.05, **P value ≤ 0.01, ***P value ≤ 0.001, ****P value ≤ 0.0001. AU, arbitrary units.

Because CFH, FHR1, and FHR3 bind to MDA-epitopes, we wanted to assess if this would alter the recruitment and cofactor activity of CFH and consequently C3b deposition on MDA-surfaces (37). First, we tested the ability of FHR1 and FHR3 to compete with CFH for binding to MDA-epitopes. Increasing concentrations of FHR1 reduced the binding of CFH to coated MDA-BSA (Fig. 6*A* and *SI Appendix*, Fig. S10*A*). A similar but less pronounced effect was observed for FHR3 (Fig. 6*B* and *SI Appendix*, Fig. S10*B*).

To determine if FHR1 and FHR3 also affect the cofactor activity of CFH in factor I (FI) cleavage of C3b, we performed in vitro C3b degradation assays on MDA-decorated surfaces. CFH provided cofactor activity for FI in C3b degradation as measured by the generation of C3b α-chain 43-kDa degradation products (Fig. 6*C*, lane 2 and Fig. 6*E*, lane 2). In this assay, a significantly reduced amount of C3b α-chain 43-kDa degradation products was observed when an equal mass ratio (Fig. 6*C*, lane 5 and Fig. 6*D*) as well as when 1:2 and 1:1 molar ratios of CFH and FHR1 (Fig. 6*C*, lanes 7 and 9, respectively) were used. All mass and molar ratios of CFH to FHR1 used are in agreement with the majority of physiological ratios reported (36, 38–42). Independent of the concentrations used, cofactor activity for FI was not observed for FHR1 alone (Fig. 6*C*, lanes 4, 6, and 8) (43). In contrast to FHR1, FHR3 did not prevent C3b α-chain degradation in these CFH cofactor assays (Fig. 6*E*, lanes 5 and 7 and Fig. 6*F*), and FHR3 alone did not display cofactor activity for FI (Fig. 6*E*, lanes 4 and 6) (44). Due to the reduced inactivation of C3b on MDA-surfaces when CFH is displaced by FHR1, an increased deposition of C3b is expected, which would lead to enhanced complement activation. Indeed, using del/del *CFHR3* and *CFHR1* serum, we could show increased deposition of C3b on MDA-surfaces in a dose-dependent manner when this serum was supplemented with increasing concentrations of FHR1, before adding it to the MDA-coated wells (Fig. 6*G*). In addition, we could show enhanced deposition of active C3b (110-kDa fragment) and of active factor B (Bb) on MDA-coated wells from del/del *CFHR3* and *CFHR1* serum when exogenously added FHR1 is prebound to MDA-coated surfaces (*SI Appendix*, Fig. S10*C*, lane containing FHR1+serum and *SI Appendix*, Fig. S10 *D* and *E*). These data demonstrate enhanced C3 convertase formation when FHR1 is bound to MDA-epitopes. No effect on C3b and Bb deposition was seen using the same del/del *CFHR3* and *CFHR1* serum when MDA-coated wells were bound by FHR3 (*SI Appendix*, Fig. S10*C*, lane containing FHR3+serum and *SI Appendix*, Fig. S10 *D* and *E*). These differential effects of FHR1 and FHR3 are likely a result of the different affinities for MDA-epitopes. Thus, FHR1 has the ability to directly compete and interfere with the binding of CFH to MDA-epitopes and thereby allows for unregulated C3 complement activation on MDA-decorated surfaces.

## Discussion

Using GWAS, we identified two major genetic variants (namely, rs1061170 and deletions of the *CFHR3* and *CFHR1* loci) that irrespectively affect CFH binding to MDA-BSA in plasma of 1,830 Caucasians of German origin with and without MDD. After controlling for age, sex, case-control status, and ethnicity, both variants had genome-wide significance (1.45 × 10^−40^ and 2.53 × 10^−22^, respectively), and their relevance was observed in a subcohort of healthy individuals only. We could show that each copy of the minor allele C of rs1061170 decreased CFH/FHL-1 binding to MDA-epitopes, as previously demonstrated in a cohort of AMD patients (14). Moreover, we revealed that CNVs, primarily deletions in *CFHR3* and *CFHR1*, alter the ability of CFH/FHL-1 in plasma to bind MDA-surfaces and that purified FHR1 and FHR3 directly compete with CFH for binding to MDA-epitopes. The importance of this interaction is supported by the fact that FHR1 and—to a lesser extent—FHR3 also bound to MDA-enriched areas on necrotic cells. One functional consequence of this interaction was revealed by the fact that FHR1 impaired the ability of CFH to provide cofactor activity for FI on MDA-decorated surfaces, resulting in enhanced deposition of C3b that can lead to propagation of the complement cascade.

The *CFH* gene is located on chromosome 1q31.3, in a 360-kbp locus of the so-called *CFH-CFHR* gene cluster. This cluster, which originated from incomplete genomic duplications of exons coding for CFH domains SCR6-8 and SCR18-20, contains long dispersed repeat elements (34). Due to this, the *CFH-CFHR* gene cluster is genetically instable, and all six proteins within it share very high sequence homology (34, 45). In addition, various genetic studies have identified this locus as a region enriched with common and rare mutations associated with susceptibility to AMD, aHUS, C3G, IgA nephropathy, and SLE (45). One of the best studied genetic variations within the *CFH* is rs1061170, a nonsynonymous SNP, which results in the exchange of T to C in exon 9 and leads to substitution of tyrosine to histidine in position 402 (SCR7) in CFH. Studies have shown that the Y402H polymorphism, a risk factor for AMD development, is associated with diminished binding of CFH and FHL-1 to GAGs and CRP (46–49). Importantly, we have previously identified MDA as a ligand for CFH and demonstrated in 171 AMD patients that the presence of the rs1061170 minor allele C decreases binding of CFH to MDA (14), which provided a mechanistic explanation for the strong disease association of this SNP, as AMD is associated with increased oxidative stress. However, it was unknown if any other genetic variant within *CFH* could also affect the binding to MDA-epitopes, as suggested by in vitro studies using 12 recombinant short proteins of CFH consisting of domains SCR19-20 and carrying 12 most common aHUS-predisposing mutations (50). This study showed that eight of 12 examined aHUS-associated mutations within the CFH C terminus alter the ability of these CFH proteins to bind to MDA and that SCR6-8 and SCR19-20 bind to MDA in a different manner. However, in the present study, we have confirmed rs1061170 as the major genetic modulator of CFH/FHL-1 binding to MDA-surfaces, independently of age and sex. Interestingly, in our assay none of the common variants within the C terminus influenced plasma CFH binding to MDA in a significant manner, which clearly supports the importance of SCR7 in recruitment of CFH to altered-self surfaces and homeostasis maintenance.

Apart from rs1061170, we have shown that the second most significant modulator of CFH binding to MDA-BSA was rs371960809, an intergenic variant with hitherto unknown significance. This variant is located downstream of the *CFH*, within the region containing break points for rearrangements and CNVs affecting *CFHR* genes. Association of these CNVs (del) detected by MLPA with CFH/FHL-1 binding to MDA-BSA revealed that carriers of homozygous and heterozygous deletions of both *CFHR3* and *CFHR1* exhibit increased binding of CFH/FHL-1 to MDA-epitopes. In our cohort of healthy individuals, rs371960809 was in high LD with the deletion of *CFHR3* and *CFHR1* and as such serves as a proxy for this CNV. Consistent with this, carriers of the rs371960809 minor allele T, and therefore at least heterozygous for the *CFHR3* and *CFHR1* deletion, have elevated binding of CFH/FHL-1 to MDA-BSA. Furthermore, our MLPA analysis uncovered 16 additional SNPs (rs16840522, rs371960809, rs115094736, rs58175074, rs149369377, rs16840639, rs7519758, rs60642321, rs7542235, rs12144939, rs2064456, rs35253683, rs6677460, rs6677604, rs6689009, and rs731557) associated with *CFHR3* and *CFHR1* deletions among the top hits that modulate binding of CFH/FHL-1 to MDA-BSA. All these data suggested that MDA-epitopes are ligands of FHR1 and FHR3, which was further confirmed by our in vitro experiments using serum and plasma, as well as recombinant proteins. Apart from gene copy numbers, levels of circulating FHR1 and FHR3 also depend on additional factors. Levels of FHR1 depend on the abundance of circulating homodimers and, to a lesser extent, heterodimers with FHR2 (45) while levels of FHR3 depend on its allele status as it was shown that the presence of the CFHR3*B allele increases its levels (28, 51). In our study, we did not analyze the effects of FHR1 homo- or heterodimers on the interaction of CFH and MDA-BSA, and we did not detect different *CFHR1* (52) or *CFHR3* alleles (51) with distinct expression properties.

Recent work described the presence of FHR1 on necrotic and pyroptotic cells where it was found to trigger inflammation in a C3b-independent manner (36). Our study confirmed binding of FHR1 and FHR3 to MDA-epitopes on necrotic cells. In addition, we showed that the patterns of surface-bound FHR1 and FHR3 on necrotic cells are distinct: FHR1 colocalizes exclusively to the areas of MDA accumulation while FHR3 displays a more diffuse pattern. These data suggest that there might be additional ligands for FHR3 on the surface of necrotic cells. Moreover, we have shown that FHR1 and FHR3 compete with CFH for the binding to MDA-covered surfaces. For FHR1, this property results in important functional consequences as FHR1 was found to reduce CFH-mediated C3b degradation by FI on MDA-surfaces, resulting in increased deposition of C3b that is required for the formation of the alternative pathway convertase and subsequent complement activation. Interestingly, FHR3 did not have such an effect, even when added in the same concentration as CFH, an effect that can be explained by the fact that FHR1 possesses 2.8 times higher affinity toward MDA-BSA when compared to CFH. CFH can be found in plasma at concentrations of 100 to 400 µg/mL, and FHR1 represents the most abundant FHR protein in plasma, with concentration of around 100 µg/mL (reported range 30 to 130 µg/mL) (36, 38–40, 42, 45). Moreover, FHR1 predominantly occurs as homodimer in the circulation, which needs to be considered when characterizing its function. In contrast, it is assumed that FHR3 circulates in plasma at concentration of only around 1 µg/mL (45). Thus, competition of FHR1 with CFH for surface binding (e.g., on damaged cells carrying MDA-epitopes) may have several functional consequences. This competition might be more pronounced locally, especially in tissues where CFH and FHR1 concentrations differ from the ones in plasma, due to different accessibility by diffusion, as it has been shown for CFH vs. FHL-1 in the eye (53). In the future, it will be of interest to study in more depth the systemic and local consequences caused by a combination of a specific rs1061170 CFH genotype together with CNVs (del) of *CFHR1* and/or *CFHR3*.

Our study represents a cross-sectional, one-time-point analysis which did not encompass follow-up of included individuals. Therefore, we could not further prospectively study the causal effect of a certain “MDA complotype”: i.e., combination of genetic variants that determine MDA binding, on the development of complementopathies, age-related diseases, and chronic inflammatory diseases. Nevertheless, our in vitro data offer a functional explanation for diseases where deletion of *CFHR3* and *CFHR1* is protective, such as AMD, and link it to a regulatory function of the CFH and FHL-1. In immunoprivileged and MDA-rich tissues, such as the retina, FHR1 would efficiently compete with CFH or FHL-1 for binding to MDA-epitopes and therefore may disable complement regulation and accelerate disease development. In the absence of FHR1 and FHR3, this capacity of CFH/FHL-1 is not impaired. This was further supported by the impact of rs371960809 and rs6677604, two proxy SNPs for *CFHR3* and *CFHR1* deletion, on CFH/FHL-1 binding to MDA-BSA.

In summary, our unbiased approach demonstrates the importance of the combination of genetic variants within the *CFH*/*CFHR3*/*CFHR1* locus in the recognition of altered-self surface ligands, represented here by the oxidation-specific MDA-epitopes. Our data suggest that this specific “MDA complotype,” which includes rs1061170 and several proxy SNPs for *CFHR3* and *CFHR1* deletion, might be used as a stratification tool for risk assessment in a healthy population for the development of diseases associated with increased oxidative stress and aging.

## Materials and Methods

The study population included in the GWAS is described in detail in Lucae et al. (23). The study was approved by the Ethics Committee of the Ludwig Maximilians University in Munich, Germany, and an informed consent was signed by all participants. Data were collected according to the Declaration of Helsinki principles. Additional information regarding the study cohort is given in *SI Appendix*, *SI Materials and Methods*. In our assays, we used OSEs as antigens, which were generated as previously reported (14, 20, 35) and are described in more detail in *SI Appendix*, *SI Materials and Methods*. MAA-BSA is enriched in the immunogenic and more advanced condensation products of MDA and is designated everywhere as MDA-BSA. Details on ELISAs for CFH/FHL-1 binding to MDA-BSA, for quantification of CFH/FHL-1, for binding of FHRs to OSEs, and for competition between CFH and FHR1 and FHR3 in binding to MDA-BSA are provided in *SI Appendix*, *SI Materials and Methods*. Furthermore, standard protocols were used for SNP and copy number genotyping, sodium dodecyl sulfate/polyacrylamide gel electrophoresis (SDS/PAGE), immunoblotting, cofactor activity assays, C3b and factor Bb deposition assays, microscale thermophoresis, and immunofluorescence imaging, as explained in *SI Appendix*, *SI Materials and Methods*.

### Data Availability Statement.

The datasets from the GWAS analysis of CFH binding to MDA-epitopes and CFH concentration, as well as the list of SNPs correlating with deletion of *CFHR3* and *CFHR1*, are available in Datasets S1–S7. All other data discussed in the paper will be made available to readers upon request.

## Supplementary Material

Supplementary File

Supplementary File

Supplementary File

Supplementary File

Supplementary File

Supplementary File

Supplementary File

Supplementary File
