# Supplementary material for: A genome-wide association study identifies key modulators of complement factor H binding to malondialdehyde-epitopes
Source: Proc Natl Acad Sci U S A. 2020 Apr 22;117(18):9942–51. doi: 10.1073/pnas.1913970117 (PMC7211993; doi:10.1073/pnas.1913970117)
Supplement: Supplementary File [file pnas.1913970117.sapp.pdf]

PNAS
www.pnas.org

Supplementary Information for

A GENOME-WIDE ASSOCIATION STUDY IDENTIFIES KEY MODULATORS OF COMPLEMENT FACTOR H BINDING TO MALONDIALDEHYDE-EPITOPES

Lejla Alic[1] and Nikolina Papac-Milicevic[1]*, Darina Czamara, Ramona B. Rudnick, Maria Ozsvar-Kozma, Andrea Hartmann, Michael Gurbisz, Gregor Hoermann, Stefanie Haslinger-Hutter, Peter F. Zipfel, Christine Skerka, Elisabeth B. Binder and Christoph J. Binder*

Correspondence
*Christoph J. Binder, MD, PhD
Lazarettgasse 14, Bauteil 25.2/6, 1090 Vienna, Austria
Email: christoph.binder@meduniwien.ac.at

*Nikolina Papac-Milicevic, PhD
Lazarettgasse 14, Bauteil 25.2/6, 1090 Vienna, Austria
Email: nikolina.papac@meduniwien.ac.at

Author Contributions
[1]L.A. and N.P.-M. contributed equally to this article

This PDF file includes:

Supplementary text
Figures S1 to S10
SI References

www.pnas.org/cgi/doi/10.1073/pnas.1913970117

Supplementary Information Text

Supplementary Subjects, Materials and Methods

Subjects and Materials

Study population

The study population included in the genome-wide association study (GWAS) is described in detail in Lucae *et al.* (1). In brief, Caucasian patients older than 18 years with recurrent unipolar depression were recruited at the Max Planck Institute of Psychiatry in Munich and psychiatric hospitals in Augsburg and Ingolstadt. Inclusion criteria were at least two moderate-to-severe depressive episodes, while patients with manic or hypomanic episodes, mood incongruent psychotic symptoms, intravenous drug abuse and secondary depressive symptoms were excluded. The mean age of patients was 49.35±14.09 years (males: 48.49±13.57 years and females: 49.86±14.38 years). Ethnicity-, gender- and age-matched controls were recruited at the Max Planck Institute of Psychiatry. Individuals negative for lifetime mood- and anxiety disorders and schizophrenia were included in the study as controls. The study was approved by the Ethics Committee of the Ludwig Maximilians University in Munich, Germany, and an informed consent was signed by all participants.

Antigens

In our assays we used following antigens: malondialdehyde-acetaldehyde-modified bovine serum albumin (MAA-BSA), phosphorylcholine-modified BSA (PC-BSA), 4-hydroxynonenal-modified BSA (4-HNE-BSA), copper sulfate–oxidized low-density lipoprotein (CuOx-LDL) and malondialdehyde-modified LDL (MDA-LDL). PC-BSA was purchased from Biosearch Technologies, Inc., CA, USA and the rest of the antigens were in-house generated.
MAA-BSA is enriched in the immunogenic and more advanced condensation products of MDA and is designated everywhere as MDA-BSA. Shortly, 2 mg/mL of BSA were modified by 0.1 M MDA (Sigma Aldrich, St. Louis, Missouri, USA) in the presence of 0.2 M acetaldehyde (Sigma Aldrich, St. Louis, Missouri, USA) for 3 hours at pH 4.8 and 37°C. To remove unbound components, dialysis was performed using phosphate-buffered saline (PBS). Degree of modification was determined, and the amount of specific MAA fluorescent adducts present was measured with spectrophotometer at λ_{max}, emission 462 nm (λ_{ex} 394 nm). The quality of the modification was determined by immunoassays with the MDA-specific antibodies LR04 and E014. To generate 4-HNE-BSA, 2 mg of BSA were modified with 5 µmol 4-HNE (Avanti Polar Lipids, Alabaster, Alabama, USA) in PBS at pH 9.0 for 24 hours at 37°C. For maintenance of reducing conditions, 20 mM $CNBH_3$ was added. Afterwards, dialysis using PBS at pH 7.4 was performed to remove unbound components. Quality control of the modification reaction was performed by immunoassay using the NA59, a 4-HNE reactive antibody, and the extent of modification was verified.
CuOx-LDL and MDA-LDL were generated as described previously (2, 3).
The degree of modification for each antigen generated was assessed by the 2,4,6-trinitrobenzenesulfonic acid (TNBS) assay (4). In all assays where these antigens were used sham-treated BSA served as a negative control.

Cell lines

For binding of FHR1 and FHR3 to surface of necrotic cells, we used human umbilical vein endothelial cells (HUVECs, CRL-1730, American Type Culture Collection, Manassas, Virginia, USA).

Methods

Genotyping and genotype imputation

DNA was isolated from fresh EDTA blood using the Puregene® whole blood DNA-extraction kit (Gentra Systems Inc., Minnesota, USA). SNP genotypes were assessed using Illumina's 550k genotyping array. After genotype calling, only individuals with a SNP callrate > 98% and SNPs with a callrate > 98%, a minor allele frequency > 5% and in Hardy-Weinberg-Equilibrium (HWE) (*P* value > 1e-06) were kept in the analysis. Missing genotypes were imputed using shapeit2 (http://mathgen.stats.ox.ac.uk/genetics_software/shapeit/shapeit.html) and impute2 (https://mathgen.stats.ox.ac.uk/impute/impute_v2.html). The 1000 Genomes Phase III sample was used as reference set. After imputation, only SNPs with an info score of at least 0.8 were kept in the analysis. Genotype probabilities were converted into best-guessed genotypes using a threshold of 0.9. We reran quality controls on the best guessed genotypes as described above. The final dataset contained 4,278,941 SNPs, 796 controls and 728 cases. Afterwards, we ran a multi-dimensional scaling (MDS) analysis on the identity by decent (IBD)-matrix of the linkage-disequilibrium (LD)-pruned genotypes. The first two MDS-components were included as covariates in the statistical analysis to correct for possible population stratification effects. Genomic regions and gene locations are annotated according to GRCh37.p13.

ELISA for CFH/FHL-1 binding to MDA-BSA

Binding of CFH and FHL-1 to MDA-BSA was measured by chemiluminescent enzyme-linked immunosorbent assay (ELISA). Ninety-six-well white, round-bottomed BRAND immunoGrade Microplates (BrandTech, Essex, Connecticut, USA) were coated with 1 µg/mL of MDA-BSA in PBS at +4˚C overnight. After washing with PBS plates were blocked with 1% BSA in Tris-buffered saline (TBS) for 1 hour on room temperature (RT). For optimization of assay sera or plasma samples were diluted in range from 1:50 to 1:2,000 in 1% BSA/TBS. For final measurements, plasma samples diluted 1:100 in 1%BSA/TBS were added and incubated for 2 hours on RT. After washing, a monoclonal mouse anti-CFH antibody (OX-24; Cedarlane, Burlington, Ontario, Canada) was added at 0.2 µg/mL in 1% BSA/TBS and incubated for 2 hours on RT. Unbound primary antibody was removed by washing and a secondary anti-mouse-IgG-alkaline phosphatase-labeled antibody was added (1:30,000 in 1% BSA/TBS) and incubated at RT for 2 hours. CFH and FHL-1 bound to MDA-BSA were detected by chemiluminescence using Lumi-Phos (Lumigen, South-field, Michigan, USA, 33% solution in water, 1 hour on RT in dark) on Synergy 2 plate reader (BioTek, Winooski, Vermont, USA). Washing steps were performed with ELx405 Select Deep Well Microplate Washer (BioTek, Winooski, Vermont, USA). Each sample was assayed in triplicate and data are expressed as relative light units (RLU) per 100 ms. The interplate coefficient of variation for each assay was <15%. Samples with RLU/100 ms below three times of the background RLU/100 ms were excluded from further analyses.

ELISA for CFH/FHL-1 quantification

Ninety-six-well white, round-bottomed BRAND immunoGrade Microplates (BrandTech, Essex, Connecticut, USA) were coated with a monoclonal mouse anti-CFH antibody (OX-24; Cedarlane, Burlington, Ontario, Canada) at 2 µg/mL in PBS. After washing with PBS and blocking with 1% BSA/TBS, plasma diluted 1:100,000 in 1% BSA/TBS was added and incubated for 2 hours on RT. Human purified CFH was used to construct a standard curve (Complement Technology, Tyler, Texas, USA). Next, a biotinylated polyclonal goat anti-human CFH antibody (Merck, Billerica, Massachusetts, USA) at 1:15,000 was added, and incubated for 2 hours on RT. Afterwards, alkaline phosphatase-labeled Neutravidin at 1:10,000 (Pierce, Weltham, Massachusetts, USA) was incubated for 30 minutes at RT. After washing with TBS, bound CFH and FHL-1 were detected with LumiPhos (as described above). Each sample was assayed in triplicate and data are expressed as RLU/100 ms.

Detection of *CFHR3* and *CFHR1* copy number variations

In order to detect copy number variations (CNVs) in the region of *CFHR3* and *CFHR1*, multiplex ligation-dependent probe amplification (MLPA) was performed using SALSA MLPA P236 ARMD probemix (MRC Holland, Netherlands) according to the manufacturer's instructions.

FHR1, FHR2, FHR3, FHR4, FHR5 binding to OSEs

Recombinant FHR1-5 proteins were generated as previously described (5–8). Of note, recombinant FHR1 was the FHR1*A isoform. MAA-BSA, PC-BSA, 4-HNE-BSA, CuOx-LDL, and MDA-LDL were coated onto 96-well plates at 1 µg/mL. After washing with PBS and blocking with 1% BSA/TBS for 1 hour on RT, recombinant FHR1, FHR2, FHR3, FHR4, and FHR5 were added at a concentration of 1 µg/mL in 1% BSA/TBS, and incubated for 2 hours on RT. Wells were washed with PBS, and a primary polyclonal rabbit anti-FHR1 (recognizing also FHR2, FHR4, and FHR5), and polyclonal anti-FHR3 were added at a 1:5,000 dilution in 1% BSA/TBS and incubated for 2 hours on RT. Unbound primary antibody was removed by washing with PBS and a secondary anti-rabbit-IgG-alkaline phosphatase-labeled antibody (Sigma Aldrich, St. Louis, Missouri, USA) was applied at a dilution of 1:20,000 in 1% BSA/TBS. After washing with TBS, bound secondary antibody was detected by chemiluminescence using 1:3 diluted Lumi-Phos. Each sample was assayed in triplicate and quadruplicate, and data are expressed as RLU/100 ms.

In an inverse ELISA, recombinant FHR1 and FHR3 were coated onto 96-well plates at concentration 0.1, 0.5 and 1 µg/mL in PBS overnight at +4°C. Biotinylated sham-treated BSA (BSA) and MDA-BSA were applied at concentration of 1 µg/mL in 1% BSA/TBS and incubated for 2 hours. Bound biotinylated proteins were detected with alkaline phosphatase-labeled Neutravidin (Pierce, Weltham, Massachusetts, USA) at a dilution of 1:10,000 in 1% BSA/TBS (30 minutes on RT), followed by addition of diluted LumiPhos. Each sample was assayed in triplicate and data are expressed as RLU/100 ms.

For an assay assessing serum- and plasma-derived FHR1 and FHR3 binding to MDA-BSA, 4-HNE-BSA and PC-BSA, antigens were coated onto MaxiSorp microtiter plate (Nunc, Roskilde, Denmark) at a concentration of 10 µg/mL in PBS. Afterwards, plates were washed and blocked with 2% BSA/PBS. After blocking, serum and plasma samples of +/+ *CFHR3* and *CFHR1* individuals (normal human serum=NHS, normal human plasma=NHP) as well as of del/del *CFHR3* and *CFHR1* individuals were added to the appropriate wells. Serum was added at a dilution of 10% in PBS, and plasma samples were added at dilution of 2% in PBS. Binding was detected with a specific monoclonal anti-FHR1 JHD10 (5, 9) or a polyclonal anti-FHR3 antibody (9), respectively. Next, corresponding secondary antibodies labelled with either horse radish peroxidase (HRP) (DAKO, Santa Clara, California, USA) or alkaline-phosphatase (Sigma Aldrich, St. Louis, Missouri, USA) were used. Bound proteins were visualized using TMB Plus2 substrate (Kem-En-Tec Diagnostics, Taastrup, Denmark) or LumiPhos (as described above). For TMB based assays, each sample was assayed in triplicate, and OD values were measured at 450 nm (Tecan). For LumiPhos based assays, each sample was assayed in triplicate and data are expressed as RLU/100 ms.

Competition ELISA for CFH and FHRs binding to MDA-BSA

Binding of biotinylated CFH to coated MDA-BSA in the presence of FHR1 was detected by chemiluminescent ELISA. Ninety-six-well microtiter plates (BrandTech, Essex, Connecticut, USA) were coated with MDA-BSA at a concentration of 2 µg/mL in PBS on +4°C overnight. After washing and blocking with 1% BSA/PBS, biotinylated CFH (biotinylation with EZ-Link Sulfo-NHS-LC-Biotinylation Kit, Thermo Fisher Scientific, Weltham, USA; CFH from Complement Technology, Tyler, Texas, USA) was applied at a constant concentration of 2 µg/mL together with recombinant FHR1 (Origene, Rockville, Maryland, USA) or BSA as a control at increasing concentrations (0-6.25 µg/mL). After incubation for 2 hours on RT, bound biotinylated CFH was detected with alkaline phosphatase-labeled Neutravidin (Pierce, Weltham, Massachusetts, USA) after incubation for 30 minutes on RT. Bound Neutravidin was detected by chemiluminescence using Lumiphos.

Binding of CFH to coated MDA-BSA in the presence of FHR3 was detected by colorimetric ELISA. MDA-BSA was coated onto MaxiSorp microtiter plate (Nunc, Roskilde, Denmark) at a concentration

of 10 µg/mL in PBS. After washing and blocking with 2% BSA/PBS, FHR3 or BSA were incubated with increasing concentrations (0-20 µg/mL) to CFH at constant concentration of 250 nM in PBS (Complement Technology, Tyler, Texas, USA). CFH binding was detected using the M15 monoclonal antibody (10) in combination with the corresponding HRP-conjugated secondary antiserum (DAKO, Santa Clara, California, USA). Bound CFH was visualized using TMB Plus2 substrate (Kem-En-Tec Diagnostics, Taastrup, Denmark). Each sample was assayed in triplicate and OD values were measured at 450 nm (Tecan).
Primary binding data expressed in RLU/100 ms or OD values are presented. Where appropriate, data are calculated as the ratio of RLU/100 ms or OD for a particular concentration of competitor (B) and the basal RLU/100 ms or OD where no competitor was added (B0).

SDS/PAGE and immunoblotting

For testing the specificity of the monoclonal anti-CFH antibody OX-24, a 4–15% Mini-PROTEAN® TGX™ Precast Protein Gel (Bio-Rad, Hercules, California, USA) was loaded with purified CFH (300 ng, Complement Technology, Tyler, Texas, USA), 3% CFH-depleted serum or 3% normal human serum in PBS. Separated proteins were transferred to polyvinylidene fluoride (PVDF) membranes using a Bio-Rad Semi-Dry Transfer Cell Trans-Blot SD (Bio-Rad, Hercules, California, USA). After blocking with 5% non-fat milk in PBS with 0.1% Tween 20 (PBST) membranes were incubated with the OX-24 monoclonal mouse anti-CFH antibody (Cedarlane, Burlington, Ontario, Canada) at 0.5 µg/mL overnight on +4°C. Unbound primary antibody was removed by washing with PBST and membranes were incubated for 2 hours on RT with a secondary HRP-labeled, anti-mouse IgG antibody (Cell Signaling Technology, Danvers, MA, USA) at a 1:3,500 dilution in 5% non-fat milk in PBST. Bound antibodies were detected using Amersham ECL Prime Western Blotting Detection Reagent (GE Healthcare, Little Chalfont, United Kingdom) with a BioRad ChemiDoc Imager (Bio-Rad, Hercules, California, USA).

Cofactor activity assay

MDA-BSA at a concentration of 10 µg/mL in PBS was coated to Maxisorp 96-well flat-bottom microtitration plates (Nunc, Roskilde, Denmark). Plates were washed and blocked with 1% BSA/PBS. Afterwards, 10 µg/mL (72 nM) of CFH in the presence or absence of 10 µg/mL (288 nM), 144 nM or 72 nM of FHR1, or 10 µg/mL (around 326 nM) or 72 nM of FHR3 was applied for 2 hours at RT. After washing, a mixture of factor I (FI) (Complement Technology, Tyler, Texas, USA) at 1.2 µg/mL and C3b (Complement Technology, Tyler, Texas, USA) at 1 µg/mL in PBS was incubated for 180 min at 37°C. The reaction was stopped by adding 1x Pierce™ Lane Marker Reducing Sample Buffer (Thermo Fisher Scientific, Weltham, USA) and samples were denatured at 95°C for 5 minutes. Samples were loaded on 4–15% Mini-PROTEAN® TGX™ Precast Protein Gels (Bio-Rad, Hercules, California, USA) and SDS/PAGE and transfer were performed as described above. For detection of C3b fragments blocked membranes were incubated with goat anti-human C3 antibody (Complement Technology, Tyler, Texas, USA) at a 1:2,000 dilution for 2 hours at RT and subsequently with an HRP-labeled donkey-anti-goat IgG secondary antibody (Santa Cruz, Dallas, Texas, USA) at 1:7,500 for 1 hour at RT. Signal detection and imaging was done as described above.

C3b and factor Bb deposition assay

Maxisorp 96-well flat-bottom microtitration plates (Nunc, Roskilde, Denmark) were coated with 10 µg/mL of MDA-BSA in PBS containing Ca^{2+} and Mg^{2+} (Gibco, Thermo Fisher Scientific, Weltham, USA). Afterwards, plates were washed and blocked with 1% BSA/PBS/Ca^{2+}/Mg^{2+} and incubated with or without 10 µg/mL of FHR1 or FHR3 in PBS/Ca^{2+}/Mg^{2+} for 2 hours on RT. Plates were washed and subsequently incubated with 5% complement active serum of a del/del *CFHR3* and *CFHR1* individual diluted in PBS/Ca^{2+}/Mg^{2+} for 1 hour at 37°C. Thereafter, the supernatant was removed and plates were extensively washed with PBS/Ca^{2+}/Mg^{2+}. After washing, deposited complement components on MDA-coated wells were collected by adding 1x Pierce™ Lane Marker Reducing Sample Buffer (Thermo Fisher Scientific, Weltham, USA) and samples were denatured at 95°C for

5 minutes. Samples were loaded on 4–15% Mini-PROTEAN® TGX™ Precast Protein Gels (Bio-Rad, Hercules, California, USA) and SDS/PAGE and transfer were performed as described above. For detection of C3b 110-kDa fragments, blocked membranes were incubated with a goat anti-human C3 antibody (Complement Technology, Tyler, Texas, USA) at a 1:2,000 dilution for 2 hours at RT and subsequently with an HRP-labeled donkey-anti-goat IgG secondary antibody (Santa Cruz, Dallas, Texas, USA) at 1:7,500 for 2 hours at RT. For detection of the 60-kDa factor B fragment b, blocked membranes were incubated with a rabbit anti-human factor B antibody (Abcam, Cambridge, United Kingdom) at a 1:1,000 dilution for 2 hours at RT and subsequently with ECL anti-rabbit IgG HRP-linked whole antibody (GE Healthcare, Chicago, Illinois, USA) at a dilution of 1:2,500 for 2 hours at RT. Signal detection and imaging was done as described above. Both proteins were detected and band intensity was densitometrically quantified using Bio-Rad Image Lab software.

ELISA for C3b deposition on MDA-surfaces

Recombinant FHR1 expressed as previously described (11) was added to 10% complement active human del/del *CFHR3, CFHR1* serum in a range of concentrations from 12.5 to 100 µg/mL. As a negative control 100 µg/mL of BSA was used. The supplemented serum was added to wells of an ELISA plate (Maxisorb, Nunc), coated with MAA-BSA at concentration of 10 µg/mL, and was incubated for 1 hour at 37°C. The samples were removed by aspiration and wells were washed with 2.5% BSA in PBS. After washing, a neo anti-C3b antibody was added (Quidel, San Diego, California, USA) followed by HRP-conjugated secondary anti-mouse serum (DAKO, Santa Clara, California, USA), incubated for 1 hour at RT. C3b was detected upon addition of TMB Plus2 substrate (Kem-En-Tec Diagnostics, Taastrup, Denmark). Each sample was assayed in triplicate and optical density was determined at 450 nm using a Tecan reader (Tecan, Männedorf, Switzerland).

Microscale thermophoresis

MAA-BSA was labelled using 647 RED-NHS labelling kit (Nano Temper Technologies, Munich, Germany). The fluorescently labeled MAA-BSA was used in solution at a concentration of 10 nM and the concentration of CFH, FHR1 and FHR3 was varied using dilutions of 1:2. Thermophoresis was measured at 25°C using a MonolithNT.115 instrument, in premium capillaries (Nano Temper Technologies) at 80% LED power. Data were analyzed with Affinity Analysis Software as described (12).

Immunofluorescence imaging

Human umbilical vein endothelial cells (HUVECs, CRL-1730, American Type Culture Collection, Manassas, VA, USA) were cultivated in Dulbecco's modified eagle medium (DMEM, Lonza, Wuppertal, Germany) supplemented with 10% fetal bovine serum (FBS, Sigma Aldrich), 25 mg/mL gentamicin-sulfate and 2 mM ultra-glutamin (Lonza). Necrosis was induced by heating the cells on 65˚C for 45 minutes. Necrotic cells were incubated with recombinant FHR1 or FHR3 or treated with BSA (500 nM in PBS) at 25˚C for 45 minutes. After washing, bound FHR1 or FHR3 were detected using monoclonal mouse antibody (mAb FHR1/mAb FHR3; generated in house), followed by Alexa Fluor 488 mouse-antiserum (Thermo Fisher Scientific, Darmstadt, Germany). MDA-epitopes present on the necrotic cells were detected using anti-MDA antibodies LR04 or NA17 with concentration of 10 µg/mL, followed by anti-mouse IgM antibody labeled with Alexa Fluor 488. The cell nucleus and the cell membrane were visualized by 4′,6-diamidino-2-phenylindole (DAPI, Sigma Aldrich) or wheat germ agglutinin (WGA) labeled with TexasRed (Thermo Fisher Scientific), respectively. Fluorescence was analyzed by confocal microscopy (Zeiss LSM 710 confocal microscope) and evaluated by ZEN2009 software (Zeiss, Jena, Germany).

Statistical methods

All statistical analyses were performed using Graphpad Prism (Windows version 8.0, Graphpad Software, San Diego, CA, USA). Continuous variables were reported as mean (with ± standard deviation (SD) or standard error of mean (SEM)) or median (with 25th-75th percentile), where appropriate. Categorical data were summarized as absolute frequencies (with percentages). Two groups of continuous variables were compared with unpaired t-test or Mann-Whitney *U* test. Multiple groups were compared by one-way ANOVA, Kruskal-Wallis test or two-way ANOVA, with multiple comparison by Tukey's, Dunn's or Sidak's *post hoc* test, respectively. Discrete covariates were summarized as frequencies and group percentages and compared using Pearson's Chi-square test or Fischer's exact test.
Statistical analyses for the GWAS were conducted in PLINK 1.90 (http://www.cog-genomics.org/plink2/) (13) using the option --linear to conduct linear regression analysis. Age, sex, the first two MDS-components as well as case-control status (for analysis of whole dataset) were used as covariates. Conditioned analysis on rs1061170 was conducted with the --condition flag. *P* values 5×10^{-8} were set as genome-wide significant. Correlation/LD patterns between SNP and deletion genotypes as well as Manhattan plots were created using R 3.5.2 (https://www.r-project.org) and the R-package GWASTools. LocusZoom plots were generated with the online software tool available at http://locuszoom.org.

Data availability

The datasets generated and/or analyzed during the current study are available from the corresponding author on reasonable request.

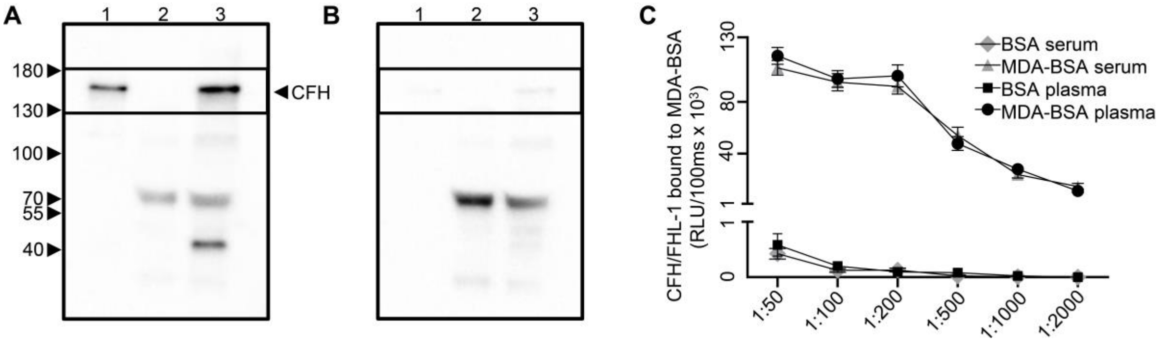

Fig. S1. Development of specific ELISA for CFH/FHL-1 binding to MDA-epitopes – confirmation of specificity of OX-24 monoclonal anti-CFH antibody and determination of optimal serum dilution. (A) Immunoblot probed with monoclonal anti-CFH antibody (OX-24). Gels were loaded with purified CFH (lane 1, 300 ng), 3% CFH-depleted serum (lane 2) and 3% healthy individual’s serum (lane 3) in PBS. (B) The stripped membrane was incubated with secondary antibody only. (C) ELISA in which different dilutions of a healthy individual’s serum and plasma (x-axis) were applied to coated MDA-BSA or sham-treated BSA (BSA). CFH/FHL-1 binding to MDA-BSA reached a plateau at a dilution between 1:50 to 1:200, thus, for our application a dilution 1:100 was used. Data represent quadruplicate determinations of three independent experiments. Values for each point are mean ± SEM values.

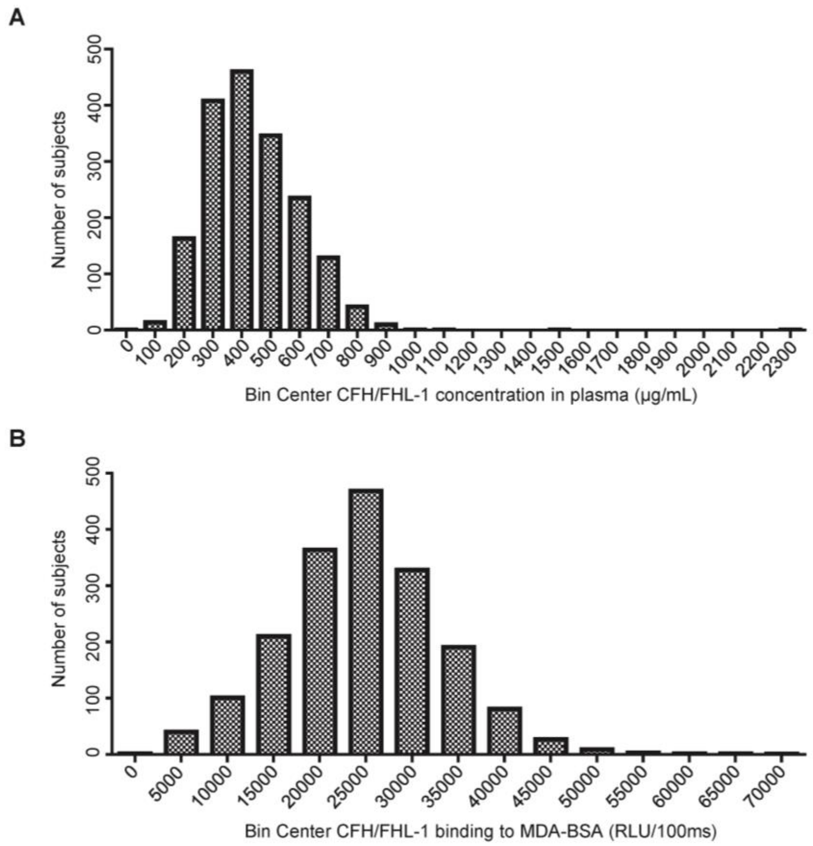

Fig. S2. Histogram of CFH/FHL-1 concentration and CFH/FHL-1 binding to MDA-BSA in plasma of study subjects (n=1,830). (A) Distribution of CFH/FHL-1 concentration in plasma (μg/mL). (B) Distribution of CFH/FHL-1 binding to MDA-BSA (RLU/100ms). Bars represent the numbers of individuals with corresponding values of measured parameters.

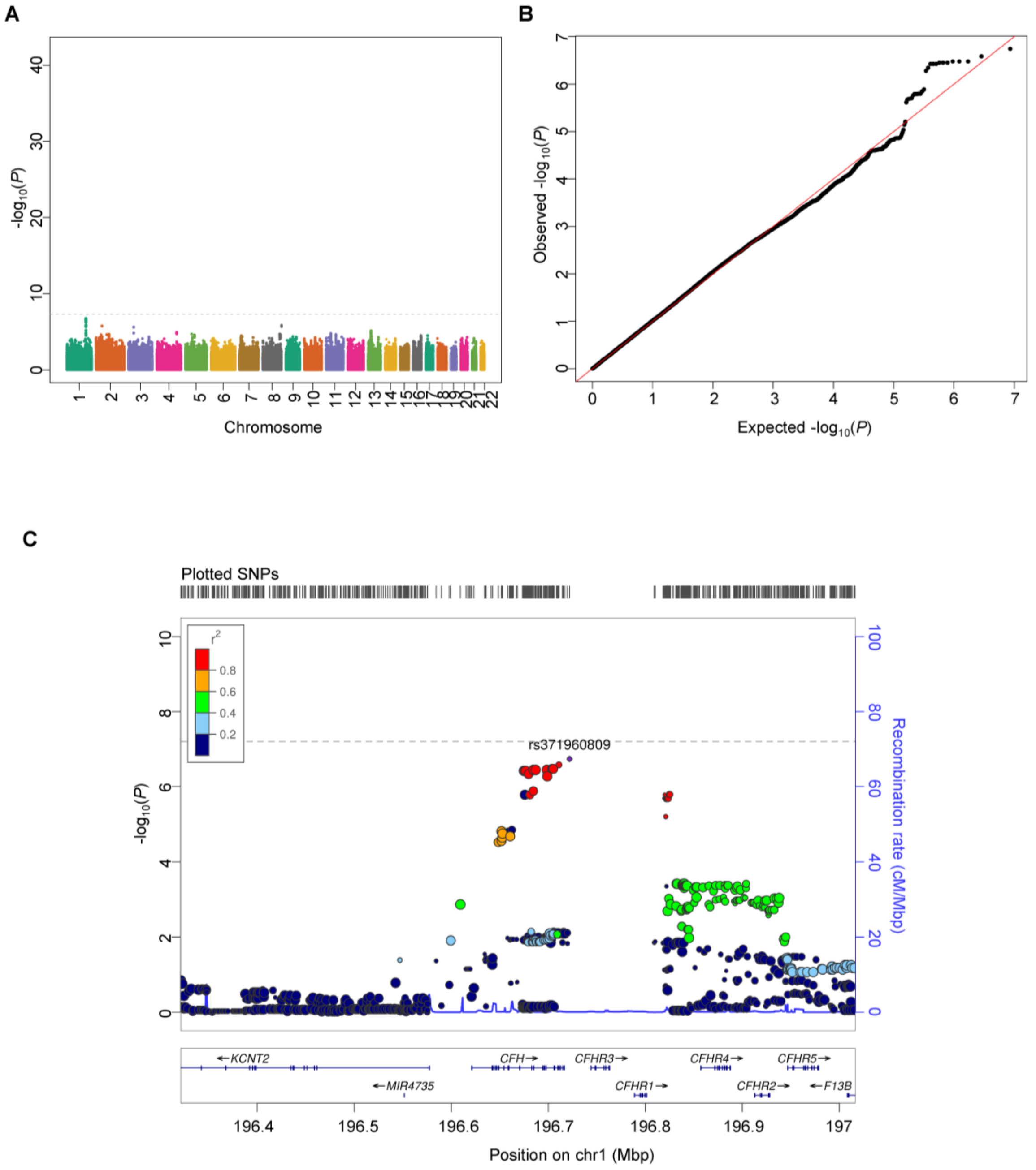

Fig. S3. GWAS of plasma CFH/FHL-1 binding to MDA-BSA in the integrated cohort (n=1,830) using age, sex, case-control status, first two principal components and rs1061170 as covariates. (A) Manhattan plot summarizing GWAS of CFH/FHL-1 binding to MDA-BSA showing $-\log_{10}(P)$ values for all SNPs. The plot includes genotyped and imputed unpruned data. The GWAS significance threshold was set at $-\log_{10}(P)=7.3$ ($P=5\times10^{-8}$) (grey dashed line). (B) QQ plot for the genotyped and imputed SNP interaction with CFH/FHL-1 binding to MDA-BSA. Observed *P* values (black dots) are plotted against the expected *P* values if no association is assumed (full red line). (C) LocusZoom plot showing unpruned SNPs associated with CFH/FHL-1 binding to MDA-BSA with its $-\log_{10}(P)$ values, at chromosomal region 1q31.3, based on analysis adjusted for age, sex, case-control status, first two principal components to correct for population stratification and rs1061170. The grey dashed line represents GWAS significance threshold. Color coding represents LD r^2 values of neighboring SNPs to the leading rs371960809. Mbp, mega base pairs.

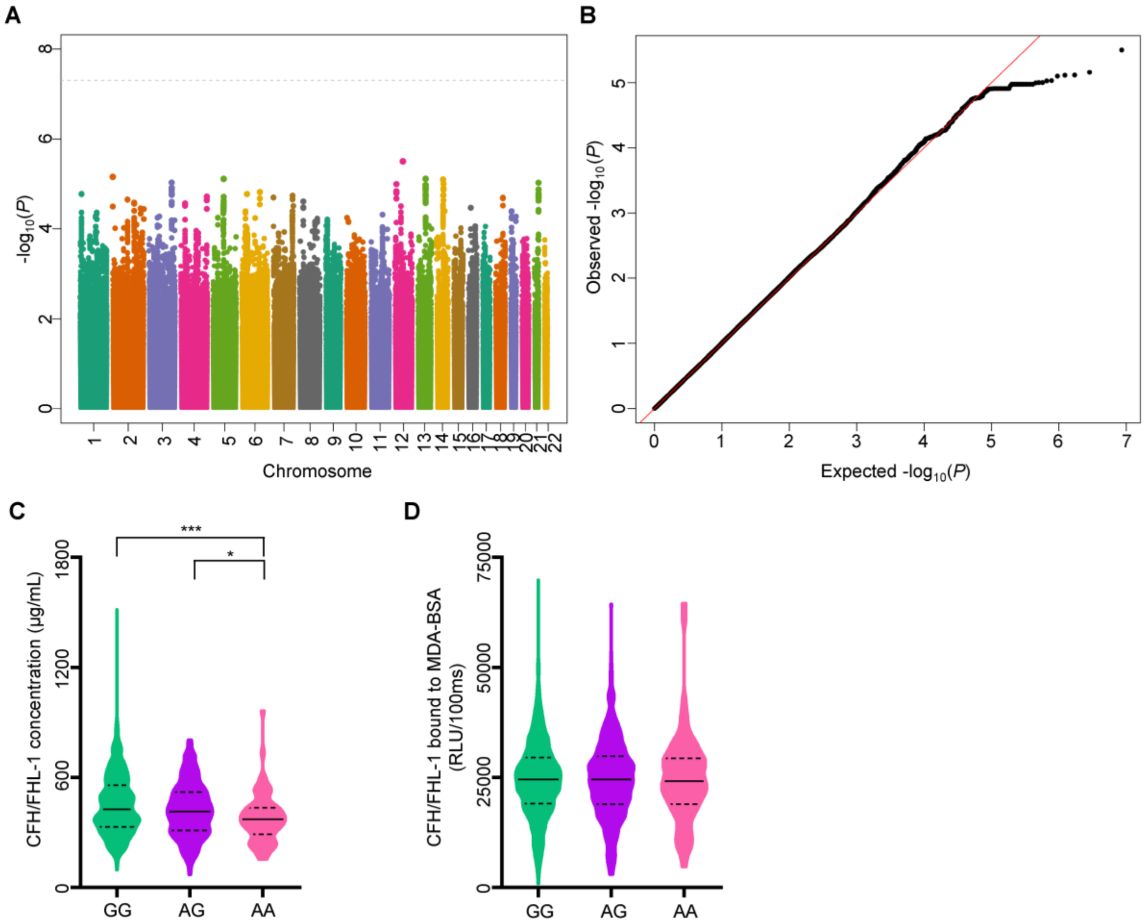

Fig. S4. GWAS of CFH/FHL-1 concentration in plasma of the integrated cohort (n=1,830). (A) Manhattan plot summarizing GWAS of CFH/FHL-1 concentration in plasma with $-\log_{10}(P)$ values for all SNPs. The plot includes current and imputed data. The GWAS significance threshold was set at $-\log_{10}(P)=7.3$ ($P=5\times10^{-8}$) (grey dashed line). The SNP with the highest $-\log_{10}(P)$ value was located in chromosome 12. (B) QQ plot for the genotyped and imputed SNP interaction with plasma CFH/FHL-1 concentration. Observed *P* values (black dots) are plotted against the expected *P* values if no association is assumed (full red line). Results are based on an analysis adjusted for age, sex, case-control status and first two principal components to correct for population stratification. (C) Association of CFH/FHL-1 concentration and rs10784193 genotype. GG, n=922; AG, n=489; AA, n=70. (D) Association of CFH/FHL-1 binding to MDA-BSA and rs10784193 genotype. GG, n=930; AG, n=494; AA, n=70. Data in (C, D) are presented as violin plot, where dashed line represents quartiles, full line represents median and width represents the number of individuals with the same value of the measured parameter. Groups compared with Kruskal-Wallis test, with Dunn's *post hoc* multiple comparison test. * *P* value ≤ 0.05, *** *P* value ≤ 0.001.

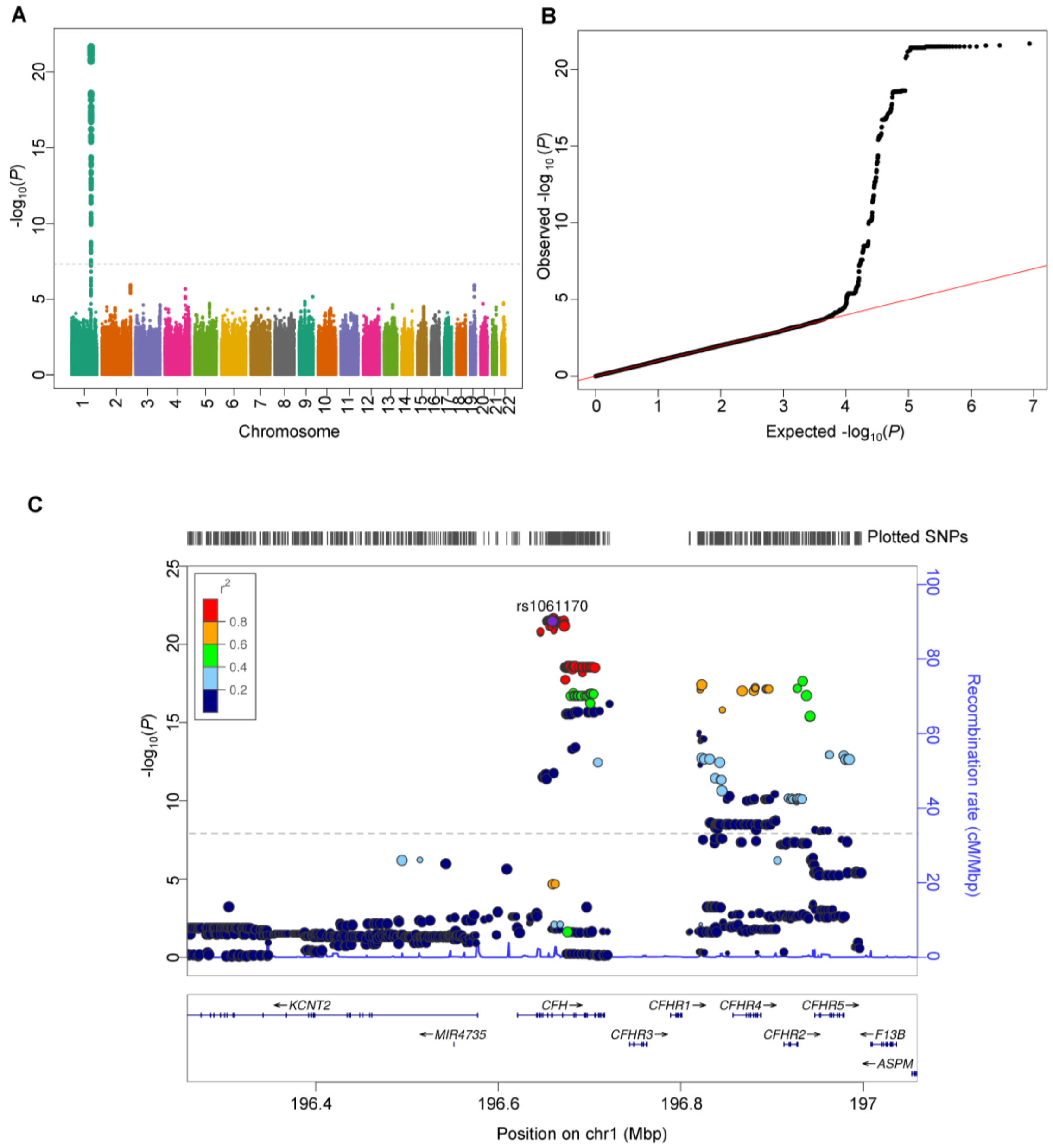

Fig. S5. GWAS of plasma CFH/FHL-1 binding to MDA-BSA in healthy individuals (n=934). (A) Manhattan plot summarizing GWAS of CFH/FHL-1 binding to MDA-BSA showing $-\log_{10}(P)$ values for all SNPs. The plot includes genotyped and imputed unpruned data. The GWAS significance threshold was set at $-\log_{10}(P)=7.3$ ($P=5\times10^{-8}$) (grey dashed line). SNPs that passed the threshold are located in 1q31.3 region. (B) QQ plot for the genotyped and imputed SNP interaction with CFH/FHL-1 binding to MDA-BSA. Observed *P* values (black dots) are plotted against the expected *P* values if no association is assumed (full red line). (C) LocusZoom plot showing unpruned SNPs with its $-\log_{10}(P)$ values at chromosomal region 1q31.3, based on an analysis adjusted for age, sex and first two principal components to correct for population stratification. The grey dashed line represents the GWAS significance threshold. Color coding represents LD r^2 values of neighboring SNPs to the leading exonic SNP rs1061170. Mbp, mega base pairs.

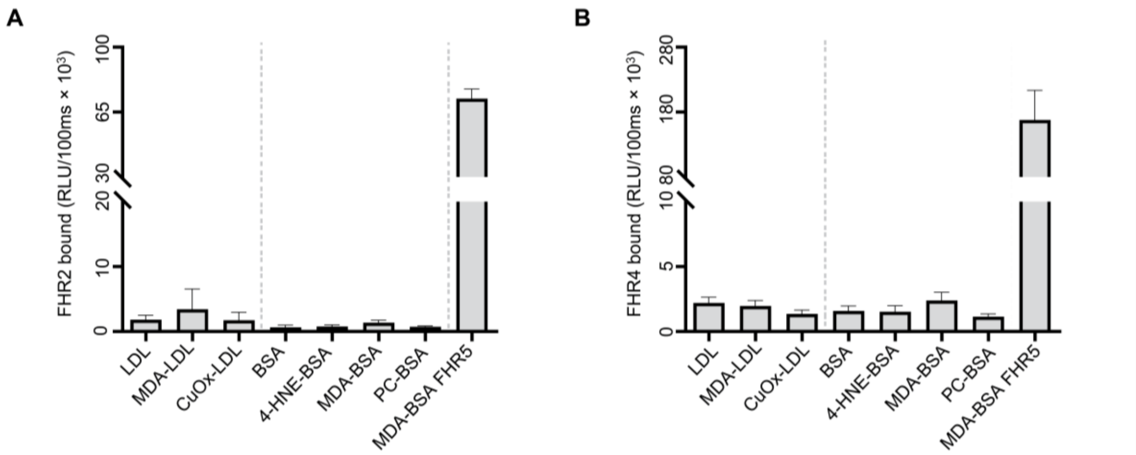

Fig. S6. Binding of FHR2 and FHR4 to MDA-epitopes. (A) Binding of FHR2 and (B) FHR4 to coated oxidation-specific epitopes (OSEs). Binding of FHR5 to MDA-BSA was used as a positive control. Bars represent mean ± SEM values of quadruplicate determinations of two experiments.

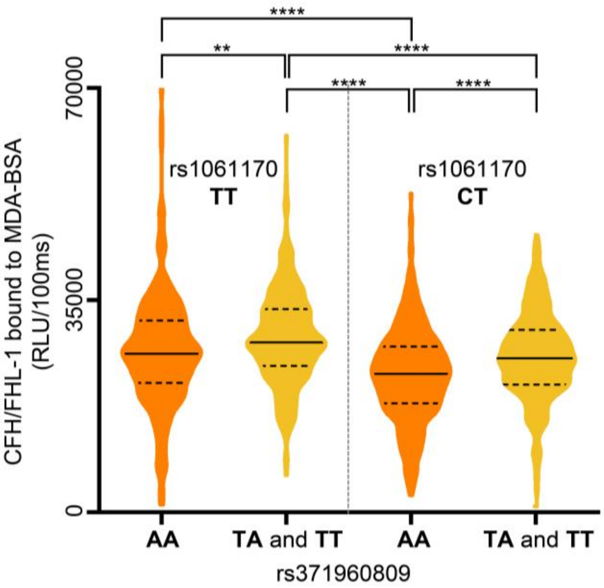

Fig S7. Association of CFH/FHL-1 binding to MDA-BSA in plasma of the integrated cohort (n=1,830) stratified according to the rs1061170 and rs371960809 genotype. Genotypes are presented as rs1061170/rs371960809; TT/AA, n=252; TT/TA and TT, n=300; CT/AA, n=476; CT/TA and TT, n=244. Data are presented as violin plots, where dashed lines represent quartiles, full lines represent median and widths represent the number of individuals with the same value of the measured parameter. Groups were compared with the Kruskal-Wallis test, with Dunn's *post hoc* multiple comparison. ** *P* value ≤ 0.01, **** *P* value ≤ 0.0001.

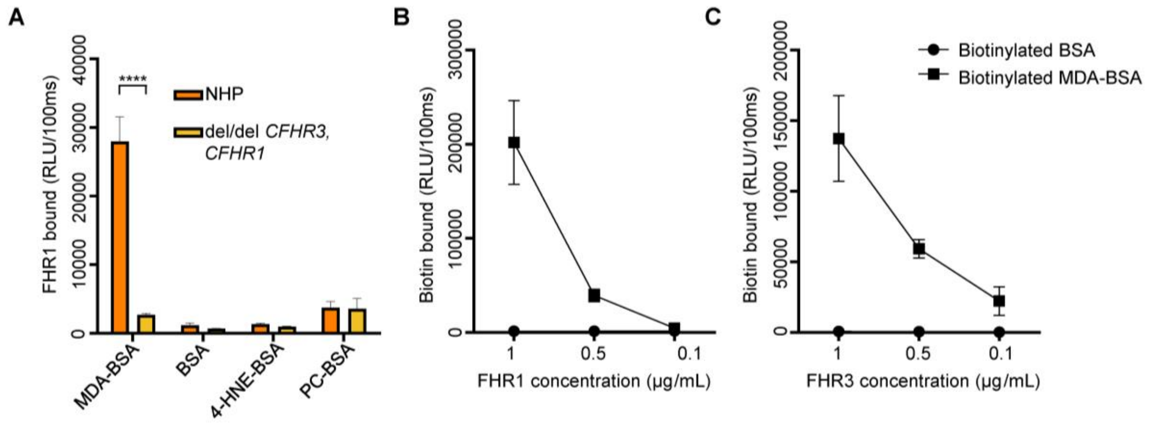

Fig. S8. Binding of FHR1 and FHR3 to MDA-epitopes. (A) Binding of plasma derived FHR1 to coated MDA-BSA, 4-HNE-BSA and PC-BSA, and sham-treated BSA (BSA). Bars represent mean ± SEM values of three healthy individuals that are +/+ *CFHR3* and *CFHR1* and three healthy individuals that are del/del *CFHR3* and *CFHR1.* All individuals carry the rs1061170 TT genotype and have similar plasma CFH concentrations (392-443 µg/mL). A representative ELISA of two is shown. (B, C) ELISA for binding of biotinylated sham-treated BSA (BSA) and biotinylated MDA-BSA to coated (B) FHR1 and (C) FHR3. Each point is represented as mean ± SEM of triplicate determinations. A. representative ELISA of three is shown. Statistical differences were tested by one-way ANOVA with Tukey‘s multiple comparison test. **** *P* value ≤ 0.0001.

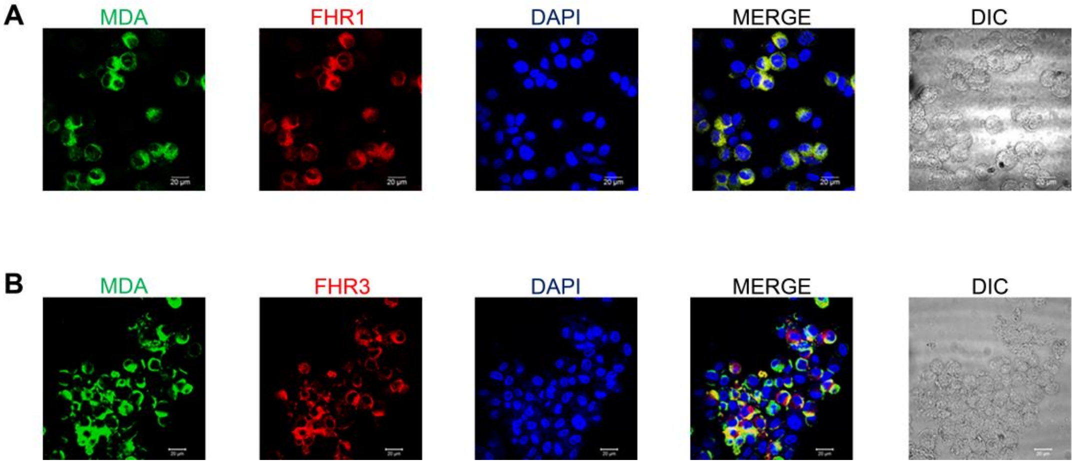

Fig. S9. Fluorescence microscopy for binding of FHR1 and FHR3 to MDA-epitopes on necrotic HUVECs. Binding of FHR1 (A) and FHR3 (B) to MDA-epitopes on necrotic HUVECs. Scale bars: 20 µm; DAPI, 4′,6-diamidino-2-phenylindole; DIC, differential interference contrast microscopy.

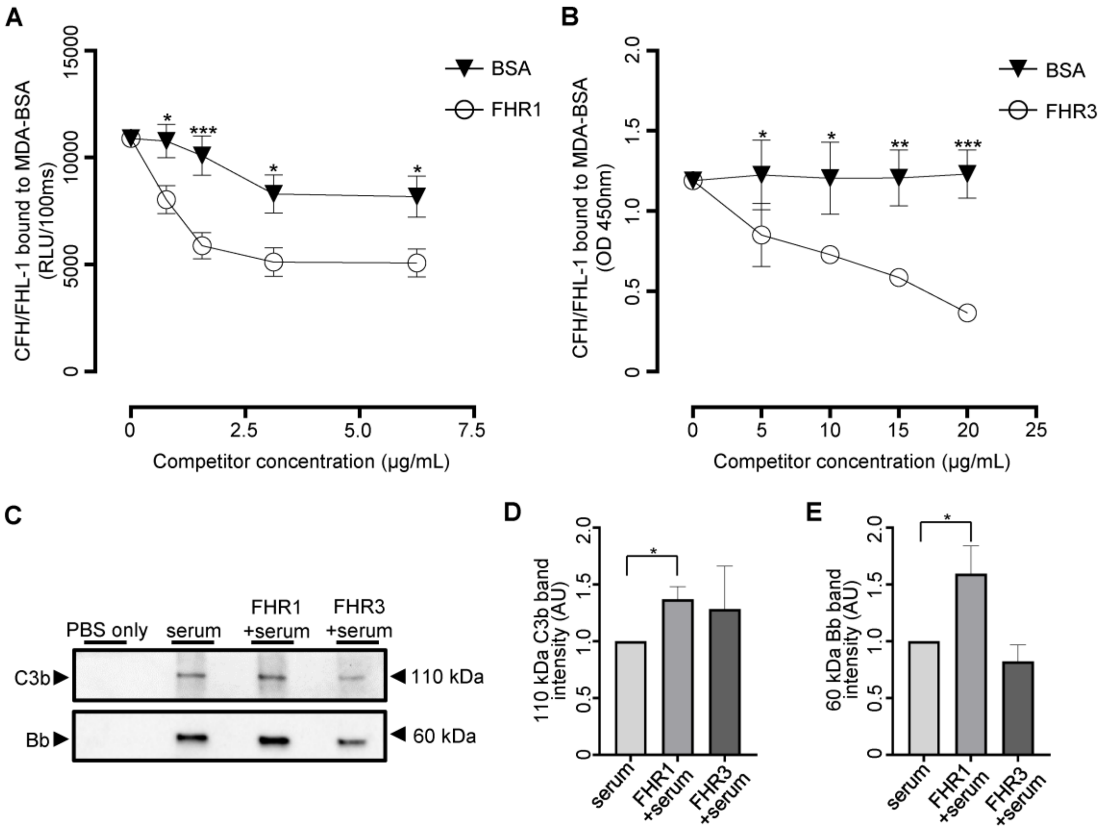

Fig. S10. Functional consequences of FHR1 and FHR3 binding to MDA surfaces. (A, B) Data shown represent the raw value (RLU/100ms) of the competition assays shown in Fig. 6 *A* and *B*. (C-E) C3b and Bb deposition assay. (C) Immunoblots show C3b and Bb fragments generated by addition of del/del *CFHR3* and *CFHR1* serum to MDA-coated wells in the presence or absence of recombinant FHR1 or FHR3, respectively. (D, E) Densitometric analysis of the band representing (D) the α-chain of active C3b and (E) Bb. Data are from two independent experiments and bars represent the mean ± SEM fold differences in density of C3b or Bb bands compared to the condition with serum only. Statistical differences were tested by two-way ANOVA with Sidak's multiple comparison (A, B) and unpaired t-test (D, E). * *P* value ≤ 0.05, ** *P* value ≤ 0.01, *** *P* value ≤ 0.001. AU, arbitrary units.

References

1. S. Lucae, et al., P2RX7, a gene coding for a purinergic ligand-gated ion channel, is associated with major depressive disorder. *Hum Mol Genet* 15(16), 2438–2445 (2006).

2. D. Weismann, et al., Complement factor H binds malondialdehyde epitopes and protects from oxidative stress. *Nature* 478(7367), 76–81 (2011).

3. S. Amir, et al., Peptide mimotopes of malondialdehyde epitopes for clinical applications in cardiovascular disease. *J Lipid Res* 53(7), 1316–1326 (2012).

4. A.F.S.A. Habeeb, Determination of free amino groups in proteins by trinitrobenzenesulfonic acid. *Anal Biochem* 14(3), 328–336 (1966).

5. S. Heinen, et al., Factor H-related protein 1 (CFHR-1) inhibits complement C5 convertase activity and terminal complex formation. *Blood* 114(12), 2439–2447 (2009).

6. H.U. Eberhardt, et al., Human factor H-related protein 2 (CFHR2) regulates complement activation. *PLoS One* 8(11), 1–11 (2013).

7. D. Buhlmann, et al., FHR3 blocks C3d-mediated coactivation of human B Cells. *J Immunol* 197(2), 620–629 (2016).

8. Q. Chen, et al., Complement factor H-related hybrid protein deregulates complement in dense deposit disease. *J Clin Invest* 124(1), 145–55 (2014).

9. C. Skerka, Q. Chen, V. Fremeaux-Bacchi, L.T. Roumenina, Complement factor H related proteins (CFHRs). *Mol Immunol* 56(3), 170–180 (2013).

10. M. Oppermann, et al., The C-terminus of complement regulator Factor H mediates target recognition: Evidence for a compact conformation of the native protein. *Clin Exp Immunol* 144, 342–352 (2006).

11. C. Skerka, S. Kuhn, K. Gunther, K. Lingelbach, P.F. Zipfel, A novel short consensus repeat-containing molecule is related to human complement factor H. *J Biol Chem* 268(4), 2904–2908 (1993).

12. T. Hallström, et al., Conserved patterns of microbial immune escape: pathogenic microbes of diverse origin target the human terminal complement inhibitor vitronectin via a single common motif. *PLoS One* 11(1), e0147709 (2016).

13. C.C. Chang, et al., Second-generation PLINK: rising to the challenge of larger and richer datasets. *Gigascience* 4(1), 7 (2015).
